# Contributions of Biological Aging to Longitudinal Incidence and Dynamic Progression of Atrial Fibrillation: A Prospective Cohort Study

**DOI:** 10.31083/RCM47208

**Published:** 2026-03-06

**Authors:** Zhixing Fan, Xinyi Liu, Hui Wu, Chaojun Yang, Jian Yang

**Affiliations:** ^1^Department of Cardiology, The First College of Clinical Medical Sciences, China Three Gorges University, 443003 Yichang, Hubei, China; ^2^Hubei Key Laboratory of Ischemic Cardiovascular Disease, 443003 Yichang, Hubei, China; ^3^Hubei Provincial Clinical Research Center for Ischemic Cardiovascular Disease, 443003 Yichang, Hubei, China; ^4^Department of Medical Record Management, The First College of Clinical Medical Sciences, China Three Gorges University, 443003 Yichang, Hubei, China

**Keywords:** KDM-BA, PhenoAge, telomere, atrial fibrillation, multi-state model

## Abstract

**Background:**

The role of biological aging in the progression of atrial fibrillation (AF) remains unclear. Therefore, the present study aimed to investigate the influence of biological aging markers on transitions from health to AF, complications, and death.

**Methods:**

Two UK Biobank datasets were analyzed: 260,198 participants for the Klemera-Doubal method for biological age (KDM-BA) and PhenoAge analyses, and 339,603 for telomere length analyses, excluding those with AF, complications (heart failure, myocardial infarction, cerebral infarction, dementia, and arterial embolic diseases) at baseline. The present study employed a multi-state model to evaluate the associations between biological aging markers and the progression of AF. Mediation analyses were utilized to assess the role of systemic inflammation.

**Results:**

During the follow-up period, 9.51–9.67% of patients in the two datasets developed AF, among whom 17.59–17.85% progressed to complications, with 8.20–10.83% of these patients dying from AF-related complications. In comparison with Q1, Q4 of the KDM-BA and PhenoAge analyses was associated with elevated risks across transitions, particularly from baseline to AF (hazard ratios (HR): 1.09, 95% confidence interval (CI): 1.04–1.14; HR: 1.30, 95% CI: 1.25–1.35), baseline to death (HR: 1.10, 95% CI: 1.04–1.16; HR: 1.11, 95% CI: 1.06–1.16), and AF to complication (HR: 1.75, 95% CI: 1.58–1.94; HR: 1.52, 95% CI: 1.37–1.68). Moreover, Q4 of the telomere length analyses showed protective effects against AF onset (HR: 0.83, 95% CI: 0.80–0.86), progression to complications (HR: 0.78, 95% CI: 0.72–0.84), and from baseline to death (HR: 0.91, 95% CI: 0.88–0.94). Systemic inflammation was associated with up to 29.95% of these associations.

**Conclusions:**

Associations were found between biological aging markers (higher KDM-BA and PhenoAge, and shorter telomere length) and the risk of AF transitions, particularly with respect to an increased risk of AF and progression to complications. These findings underscore the importance of biological age in AF risk stratification and prevention.

## 1. Introduction

Atrial fibrillation (AF) is characterized by unorganized beating of the atria and has emerged as a significant cardiovascular epidemic. Its rising incidence and prevalence are closely linked to global population ageing and improved survival from chronic diseases [1, 2]. According to the Global Burden of Disease Study 2021, AF accounted for 4.48 million new cases, 0.34 million deaths, and 8.36 million disability-adjusted life years globally in 2021 [1]. Notably, AF is associated with severe complications including stroke, myocardial infarction (MI), heart failure (HF), and dementia, with patients typically succumbing to these complications rather than AF itself [3, 4]. For instance, in 2019, the global prevalence of AF-associated HF was 1.5 million, marking a 49.8% increase from 1990 [5]. These complications underscore the urgent need for a more profound comprehension of the factors that precipitate the onset and progression of AF.

Ageing is a well-established risk factor for AF [6, 7, 8]. Biological ageing markers, including clinical traits-based biological age, such as the Klemera-Doubal method biological age (KDM-BA) and PhenoAge, as well as molecular-level telomere length, have emerged as promising tools with which to assess biological ageing status [9, 10, 11]. Basic research suggests that biological ageing contributes to AF pathogenesis through cardiac structural and electrical remodeling [12, 13]. Observational studies have shown that biological ageing is associated with an increased risk of AF [14, 15], and other studies have found that these markers predict adverse outcomes in patients with AF [16, 17, 18]. However, the influence of biological ageing on the entire trajectory—from health to AF onset, progression to complications, and mortality—remains poorly understood.

Multi-state models (MSMs) provide an advanced framework for analysing longitudinal disease progression, incorporating multiple disease states and transitions while accounting for competing risks [19]. Unlike traditional survival models, MSMs allow the simultaneous evaluation of transitions, such as from health to AF onset, AF to complications, and complications to mortality [20]. This methodology has been widely applied to study disease progression patterns, such as the trajectory from pre-diabetes to cardiovascular disease [19], and the dynamic progression of cardio-renal-metabolic multimorbidity [21]. Applying MSMs to AF progression provides a more comprehensive understanding of the impact of risk factors on various disease transitions [22].

The aim of this study was to evaluate the associations between biological ageing and AF progression, complications, and mortality using the UK Biobank cohort. MSMs were employed to explore the dynamic effects of ageing on transitions from health to AF, the development of complications (HF, MI, stroke, and dementia), and ultimately mortality.

## 2. Materials and Methods

### 2.1 Study Population

This study utilized data from the UK Biobank, which is a large-scale prospective cohort study comprising around 500,000 participants aged 40–69 years, who were recruited from 22 UK assessment centers between 2006 and 2010. Ethical approval was granted by the North West Multicenter Research Ethics Committee (REC reference: 21/NW/0157), and written informed consent was obtained from all participants. This study’s project approval number is 170605.

**Fig. 1.**
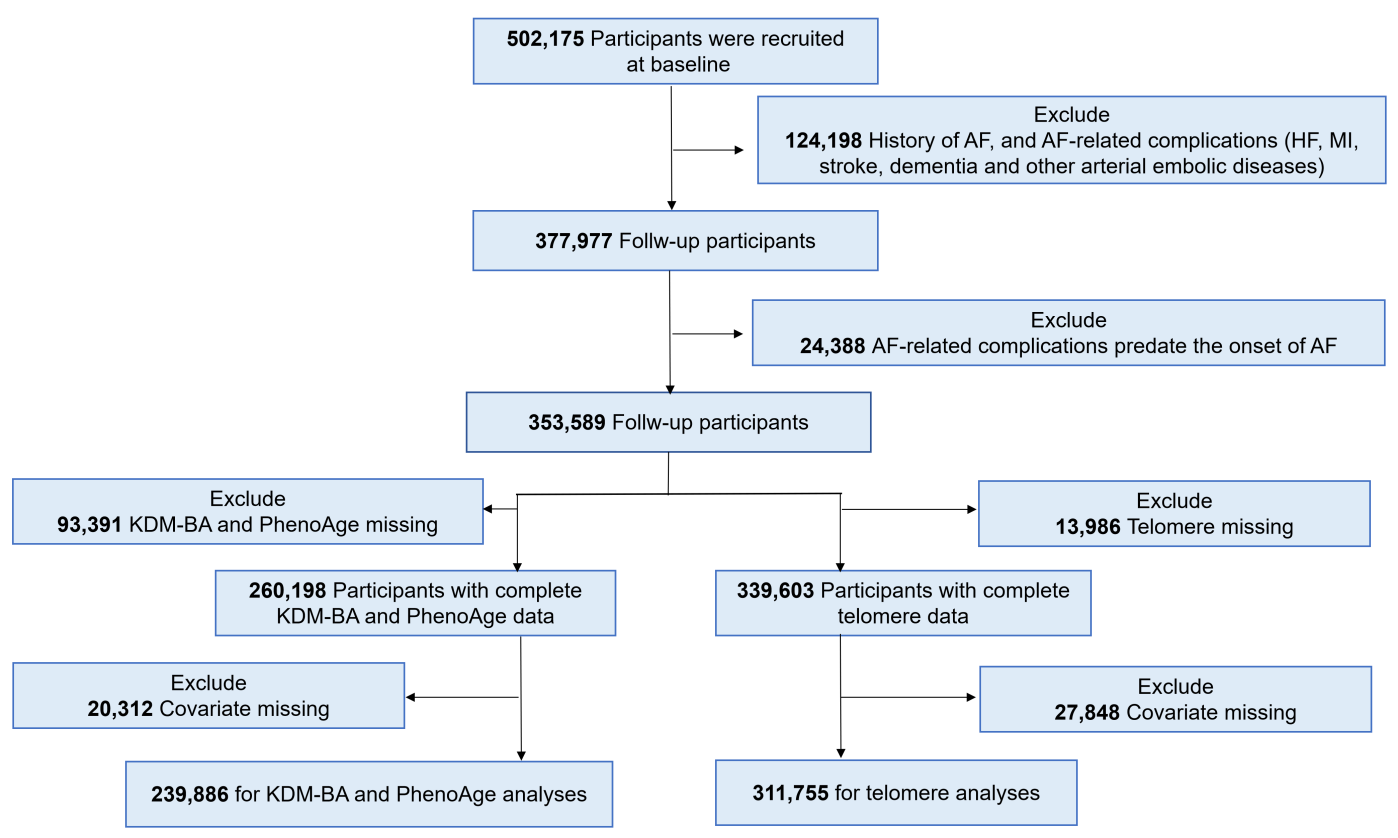
**Flowchart of participants included in this study**. AF, Atrial fibrillation; HF, Heart failure; MI, Myocardial infarction; KDM-BA, Klemera-Doubal method biological age.

Of the initial 502,175 participants, we excluded those with a history of AF or AF-related complications at baseline (n = 124,198). We also excluded participants whose AF-related complications predated AF onset (n = 24,388). For analyses involving KDM-BA and PhenoAge, we excluded participants with missing data on these measures (n = 353,589) leaving 260,198 participants for analysis. For telomere length analyses, participants with missing telomere data (n = 13,986) were excluded, resulting in 339,603 participants (Fig. 1).

### 2.2 Assessment of Biological Ageing

Biological ageing was assessed using two approaches: clinical traits-based biological age (KDM-BA and PhenoAge) [9, 10], and molecular-level telomere length [11]. KDM-BA was derived from forced expiratory volume in one second, systolic blood pressure, and seven blood biomarkers: albumin, alkaline phosphatase, blood urea nitrogen, creatinine, C-reactive protein, glycated haemoglobin, and total cholesterol. PhenoAge was calculated using nine blood biomarkers, including albumin, alkaline phosphatase, creatinine, C-reactive protein, glucose, mean cell volume, red cell distribution width, white blood cell count, and lymphocyte proportion. To quantify the deviation between biological and chronological age, we regressed KDM-BA and PhenoAge on chronological age using natural splines with three degrees of freedom. The algorithms and R codes for these measures are available in the “BioAge” R package and in prior publications.

Telomere length was measured using a quantitative polymerase chain reaction (qPCR) assay to quantify DNA extracted from leukocytes at baseline, and was expressed as the telomere-to-single copy gene ratio (T/S ratio). The measurements were adjusted for technical parameters, log-transformed, and Z-standardized. Detailed quality control procedures have been described previously [11].

### 2.3 Follow-Up for AF, AF-Related Complications and Death

Participants without a history of AF or AF-related complications at baseline were followed from recruitment until they were lost to follow-up, died, or October 31, 2022, whichever occurred first. Outcomes of interest included incident AF, AF-related complications (HF, MI, cerebral infarction, dementia, and other arterial embolic diseases), and all-cause mortality. These outcomes were identified through linkage with death registries, primary care records, and hospital inpatient data, using diagnostic codes from the International Classification of Diseases, 9th (ICD-9) and 10th (ICD-10) revisions. Detailed diagnostic codes are provided in Table 1. The validation steps of outcome events were that atrial fibrillation occurred first, followed by complications of atrial fibrillation.

**Table 1.** **Diagnoses used for the definition of AF, AF-related complications and outcomes**.

Disease	ICD code
Atrial fibrillation	ICD-10: I48; ICD-9: 4273.
Heart failure	ICD-10: I50, I110.
Myocardial infarction	ICD-10: I21–I23, I24.1, I25.2.
Stroke (cerebral infarction)	ICD-10: I63.
Other arterial embolic diseases	ICD-10: I74, I26.
Dementia	ICD-10:
	AD: F00, F00.0, F00.1, F00.2, F00.9, G30, G30.0, G30.1, G30.8, G30.9;
	VaD: F01, F01.0, F01.1, F01.2, F01.3, F01.8, F01.9, I67.3;
	FTD: F02.0, G31.0;
	Other codes for all-cause dementia: A81.0, F02, F02.1, F02.2, F02.3, F02.4, F02.8, F03, F05.1, F10.6, G31.1, G31.8.
	ICD-9:
	AD: 331.0;
	VaD: 290.4;
	FTD: 331.1;
	Other codes for all-cause dementia: 290.2, 290.3, 291.2, 294.1, 331.2, 331.5.

ICD-9, International Classification of Diseases, 9th; ICD-10, International Classification of Diseases, 10th; AD, Alzheimer’s disease; VaD, Vascular dementia; FTD, frontotemporal dementia.

### 2.4 Mediator

Systemic inflammation was assessed using the neutrophil-to-lymphocyte ratio (NLR) and the systemic inflammation response index (SIRI). The NLR was calculated by dividing the neutrophil count by the lymphocyte count, while the SIRI was calculated by multiplying the neutrophil count by the monocyte count and dividing this sum by the lymphocyte count. Both measures were log-transformed to address skewed distributions.

### 2.5 Covariates

Baseline covariates were collected via questionnaires and interviews. Demographic factors included age, sex, ethnicity, education level, and the Townsend deprivation index. Lifestyle factors included body mass index (BMI), categorized as underweight (BMI <18.5 kg/m^2^), normal weight (BMI 18.5–24.9 kg/m^2^), overweight (BMI 25–29.9 kg/m^2^), or obese (BMI ≥30 kg/m^2^); dietary pattern (healthy or unhealthy, based on DASH diet score); smoking status (never, previous, or current); and alcohol consumption (never, previous, or current).

### 2.6 Statistical Analyses

Missing covariates were imputed using multiple imputation by chained equations, with all covariates in the model. Baseline characteristics were summarized as means (standard deviations) or frequencies (percentages) for continuous and categorical variables, respectively, across all participants and subgroups with AF or AF-related complications.

Cox proportional hazards models were employed to estimate the association between biological ageing markers and the incidence of AF, AF-related complications, and mortality. To assess the progression from a healthy state to AF and subsequent complications and death, MSMs were employed using the “mstate” R package. Five transitions were modeled: (1) baseline to AF, (2) baseline to death, (3) AF to complications, (4) AF to death, and (5) complications to death. For participants entering multiple states on the same date, the entry date of the prior state was set as 0.5 days earlier. A more detailed MSM was constructed to examine specific complications (HF, MI, stroke, dementia) involving 11 transitions. In our multi-state models, death was included as an absorbing state with dedicated transitions (e.g., baseline → death), so competing risks of death for intermediate events such as AF or complications were explicitly accounted for. Each transition had an independent baseline hazard, estimated nonparametrically, under a Markov assumption conditional on the current state and covariates. The proportional hazards assumption was assessed for each transition using Schoenfeld residuals, with no major violations detected (**Supplementary Table 1,2,3**). Model 1 was adjusted for age and sex, while Model 2 was further adjusted for ethnicity, the Townsend deprivation index, education level, BMI, dietary pattern, smoking status, and alcohol consumption.

Counterfactual mediation analysis was performed to investigate the mediating role of systemic inflammation (as measured by NLR and SIRI) in the relationship between biological ageing markers and AF-related outcomes. This method estimated the direct, indirect, and total effects, as well as the proportion mediated. The “CMAverse” R package was used to calculate hazard ratios (HRs) and 95% confidence intervals (CIs) for counterfactual effects with 1000 bootstrapped samples.

The Sensitivity analyses included: (a) excluding participants lacking covariates; (b) excluding participants with events within the first two years of follow-up; (c) excluding those with a history of cancer at baseline; (d) testing alternative age-scaling methods for biological age; (e) using a semi-Markov specification (time since entry into the current state as the time scale); (f) setting a prior state’s entry time to ±1–2 days for same-day events.

All statistical analyses were performed using R software (version 4.4.2, R Foundation for Statistical Computing, Vienna, Austria). Statistical significance was defined as *p* < 0.05.

## 3. Result

### 3.1 Characteristics of the Participants

Among the 260,198 participants analyzed for KDM-BA and PhenoAge, the mean age was 55.47 years, with 57.08% being women (Table 2). Participants were predominantly white (94.51%), with 35.15% having received a college/university education. The mean BMI was 26.86 kg/m^2^, with most participants being never smokers (57.69%) and current alcohol drinkers (93.11%). Similar characteristics were observed in the telomere length cohort (n = 339,603) (**Supplementary Table 4**). Notably, participants who developed AF or its complications were more likely to be male and less educated, and to have a higher BMI.

**Table 2.** **Baseline characteristics of study participants by incident disease states in the data set of KDM-BA and PhenoAge as exposure**.

Characteristics		Overall (n = 260,198)	AF (n = 24,750)	AF-related complications (n = 4353)
Age (years)		55.47 ± 8.10	56.74 ± 8.09	58.18 ± 8.09
Sex				
	Female	148,532 (57.08)	11,801 (47.68)	1629 (37.42)
	Male	111,666 (42.92)	12,949 (52.32)	2724 (62.58)
Ethnic				
	White	245,905 (94.51)	23,721 (95.84)	4208 (96.67)
	Other	14,293 (5.49)	1029 (4.16)	145 (3.33)
Education				
	College or university degree	91,452 (35.15)	8089 (32.68)	1140 (26.19)
	A/AS level or equivalent	30,964 (11.90)	2653 (10.72)	383 (8.80)
	O/GCSEs level or equivalent	57,350 (22.04)	5205 (21.03)	912 (20.95)
	CSEs or equivalent	14,981 (5.76)	1128 (4.56)	134 (3.08)
	NVQ/HND/HNC or equivalent	16,040 (6.16)	1741 (7.03)	376 (8.64)
	Other professional qualifications	12,907 (4.96)	1382 (5.58)	265 (6.09)
	None of the above	36,504 (14.03)	4552 (18.39)	1143 (26.26)
Townsend deprivation index		–1.49 ± 2.97	–1.52 ± 2.98	–1.38 ± 3.05
BMI (kg/m^2^)		26.86 ± 4.37	27.65 ± 4.70	28.88 ± 5.05
	Thin (<18.5)	1794 (0.69)	151 (0.61)	28 (0.64)
	Normal (18.5–24.9)	93,789 (36.05)	7404 (29.92)	922 (21.18)
	Overweight (25–29.9)	111,570 (42.88)	10,751 (43.44)	1898 (43.60)
	Obesity (≥30)	53,045 (20.39)	6444 (26.04)	1505 (34.57)
Diet				
	Healthy	134,961 (51.87)	12,906 (52.15)	2274 (52.24)
	Unhealthy	125,237 (48.13)	11,844 (47.85)	2079 (47.76)
Smoking status				
	Never	150,098 (57.69)	13,111 (52.97)	1972 (45.30)
	Previous	85,772 (32.96)	9280 (37.49)	1901 (43.67)
	Current	24,328 (9.35)	2359 (9.53)	480 (11.03)
Alcohol intake				
	Never	10,336 (3.97)	952 (3.85)	172 (3.95)
	Previous	7584 (2.91)	849 (3.43)	168 (3.86)
	Current	242,278 (93.11)	22,949 (92.72)	4013 (92.19)
PhenoAge		–11.87 ± 4.74	–11.13 ± 4.96	–9.94 ± 5.14
	Q1	65,049 (25.00)	5062 (20.45)	595 (13.67)
	Q2	65,050 (25.00)	5861 (23.68)	894 (20.54)
	Q3	65,048 (25.00)	6330 (25.58)	1148 (26.37)
	Q4	65,051 (25.00)	7497 (30.29)	1716 (39.42)
KDM-BA		–13.40 ± 14.58	–14.39 ± 15.07	–13.37 ± 15.25
	Q1	65,050 (25.00)	7043 (28.46)	1193 (27.41)
	Q2	65,048 (25.00)	6170 (24.93)	1103 (25.34)
	Q3	65,050 (25.00)	5665 (22.89)	900 (20.68)
	Q4	65,050 (25.00)	5872 (23.73)	1157 (26.58)

AF-related complications included heart failure, myocardial infarction, stroke, dementia and other arterial embolism diseases. AF, Atrial fibrillation; BMI, body mass index; KDM-BA, Klemera-Doubal method biological age; A/AS, advanced/advanced subsidiary; O/GCSEs, ordinary/general certificate of secondary education; CSEs, certificate of secondary education; NVQ, national vocational qualification; HND, higher national diploma; HNC, higher national certificate.

**Fig. 2.**
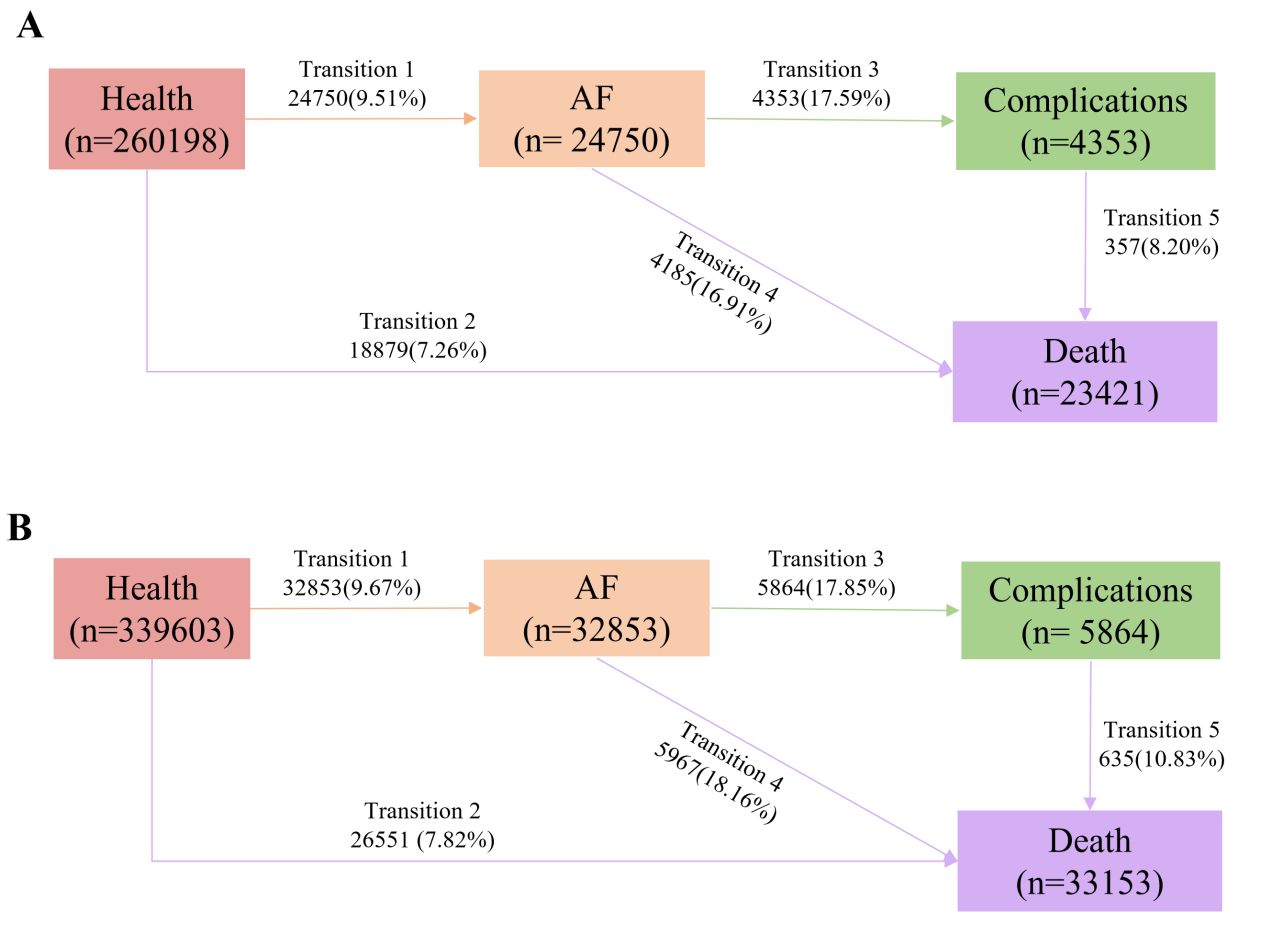
**Transitions from baseline to AF, complications, and all-cause death**. (A) Transitions in the date set of KDM-BA and PhenoAge as exposure. (B) Transitions in the date set of telomere as exposure. Complications included heart failure, myocardial infarction, stroke, dementia, and other arterial embolism diseases.

**Fig. 3.**
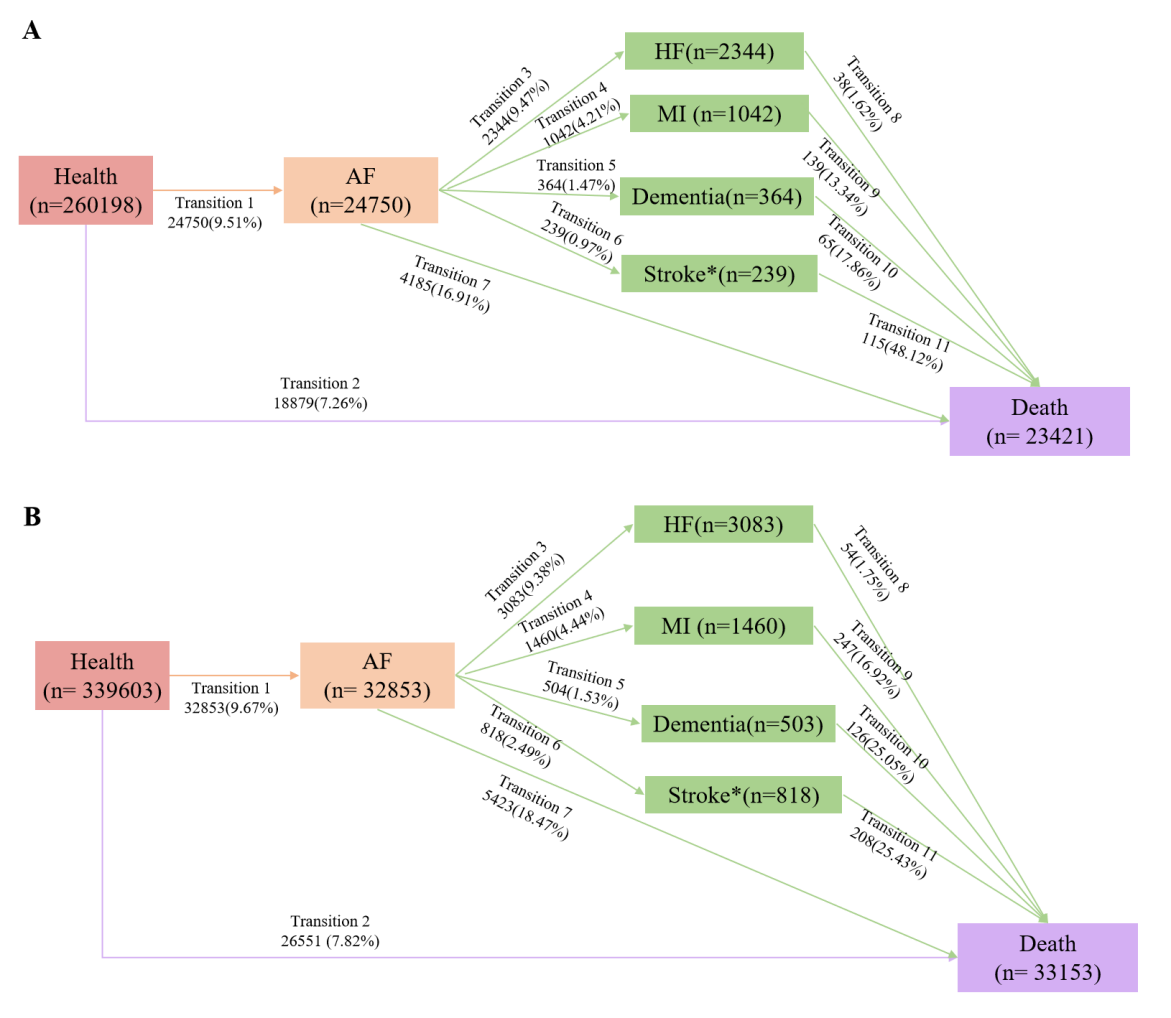
**Transitions from baseline to AF, specific complications (HF, MI, stroke, other arterial embolism disease and dementia), and all-cause death**. (A) Transitions in the date set of KDM-BA and PhenoAge as exposure. (B) Transitions in the date set of telomere as exposure. *Include the other arterial embolism disease.

During the follow-up period, 9.51% of the KDM-BA/PhenoAge cohort developed AF, with 17.59% of these cases progressing to complications. The most common complication was HF (9.47%), followed by MI (4.21%), dementia (1.47%), and stroke (0.97%) (Figs. 2,3). Of those with complications, 8.20% subsequently died. Mortality patterns revealed 7.26% deaths from baseline, 16.91% deaths after AF diagnosis, and 8.20% deaths after complications. Similar trends were seen in the telomere length cohort, where 9.67% developed AF, 17.85% of AF cases progressed to complications, and 10.83% died after complications.

### 3.2 Cox Regression Analyses

The three biological ageing markers showed significant associations with disease progression (Table 3). Compared with the lowest quartile (Q1), the highest quartile (Q4) of both KDM-BA and PhenoAge was associated with increased risks of AF (HR: 1.16, 95% CI: 1.10–1.21; HR: 1.34, 95% CI: 1.29–1.40), complications (HR: 1.25, 95% CI: 1.11–1.39; HR: 1.25, 95% CI: 1.14–1.37), and death (HR: 1.71, 95% CI: 1.62–1.82; HR: 1.87, 95% CI: 1.78–1.97). In contrast, the Q4 of telomere length showed protective effects against AF (HR: 0.76, 95% CI: 0.74–0.78), complications (HR: 0.55, 95% CI: 0.51–0.59), and death (HR: 0.90, 95% CI: 0.87–0.92).

**Table 3.** **Cox regression model to assess associations of biological ageing with AF and its state transition**.

Exposure	Model		HR (95% CI)		
			Baseline to AF	Baseline to Complication	Baseline to Death
KDM-BA					
	Model 1				
		Per score	1.01 (1.01, 1.01) *	1.01 (1.01, 1.01) *	1.02 (1.02, 1.02) *
		Q1	ref	ref	ref
		Q2	1.11 (1.06, 1.15) *	1.18 (1.07, 1.29) *	1.36 (1.30, 1.43) *
		Q3	1.21 (1.15, 1.26) *	1.25 (1.13, 1.38) *	1.57 (1.48, 1.65) *
		Q4	1.37 (1.31, 1.44) *	1.41 (1.27, 1.57) *	2.14 (2.02, 2.26) *
	Model 2				
		Per score	1.00 (1.00, 1.01) *	1.01 (1.01, 1.02) *	1.01 (1.00, 1.02) *
		Q1	ref	ref	ref
		Q2	1.03 (0.99, 1.07)	1.12 (1.02, 1.22) *	1.24 (1.18, 1.30) *
		Q3	1.08 (1.03, 1.13) *	1.15 (1.04, 1.28) *	1.35 (1.27, 1.43) *
		Q4	1.16 (1.10, 1.21) *	1.25 (1.11, 1.39) *	1.71 (1.62, 1.82) *
PhenoAge					
	Model 1				
		Per score	1.03 (1.03, 1.04) *	1.03 (1.02, 1.03) *	1.07 (1.07, 1.07) *
		Q1	ref	ref	ref
		Q2	1.12 (1.07, 1.16) *	1.06 (0.96, 1.16)	1.18 (1.12, 1.25) *
		Q3	1.24 (1.20, 1.30) *	1.16 (106, 1.27) *	1.44 (1.36, 1.51) *
		Q4	1.48 (1.42, 1.54) *	1.35 (1.24, 1.48) *	2.20 (2.09, 2.31) *
	Model 2				
		Per score	1.03 (1.02, 1.03) *	1.02 (1.01, 1.03) *	1.06 (1.06, 1.06) *
		Q1	ref	ref	ref
		Q2	1.08 (1.03, 1.12) *	1.02 (0.93, 1.12)	1.15 (1.08, 1.21) *
		Q3	1.16 (1.12, 1.21) *	1.10 (1.01, 1.21) *	1.33 (1.26, 141) *
		Q4	1.34 (1.29, 1.40) *	1.25 (1.14, 1.37) *	1.87 (1.78, 1.97) *
Telomere					
	Model 1				
		Per score	0.42 (0.38, 0.46) *	0.15 (0.12, 0.18) *	0.65 (0.59, 0.71) *
		Q1	ref	ref	ref
		Q2	0.90 (0.88, 0.93) *	0.82 (0.77, 0.87) *	0.91 (0.88, 0.94) *
		Q3	0.83 (0.81, 0.86) *	0.63 (0.58, 0.67) *	0.89 (0.86, 0.91) *
		Q4	0.75 (0.73, 0.77) *	0.54 (0.50, 0.58) *	0.87 (0.85, 090) *
	Model 2				
		Per score	0.44 (0.40, 0.48) *	0.15 (0.12, 0.19) *	0.70 (0.64, 0.76) *
		Q1	ref	ref	ref
		Q2	0.91 (0.88, 0.94) *	0.82 (0.77, 0.88) *	0.92 (0.89, 0.95) *
		Q3	0.84 (0.82, 0.87) *	0.63 (0.59, 0.68) *	0.90 (0.87, 0.93) *
		Q4	0.76 (0.74, 0.78) *	0.55 (0.51, 0.59) *	0.90 (0.87, 0.92) *

Model 1 was adjusted for sex and ethnicity; Model 2 was adjusted for sex, ethnicity, education, Townsend deprivation index, BMI, diet, smoking status, and alcohol intake. Complications included heart failure, myocardial infarction, stroke, dementia and other arterial embolism diseases. HR, hazard ratio; CI, confidence interval. *p < 0.05.

### 3.3 Multi-State Analyses

In the multistate models, all three biological ageing markers showed distinct patterns in different disease transitions (Table 4). For KDM-BA, the Q4 was significantly associated with increased risks of transitions from baseline to AF (HR: 1.09, 95% CI: 1.04–1.14), baseline to death (HR: 1.10, 95% CI: 1.04–1.16), and AF to complication (HR: 1.75, 95% CI: 1.58–1.94). Similar patterns were observed for PhenoAge, with the Q4 showing elevated risks in transitions from baseline to AF (HR: 1.30, 95% CI: 1.25–1.35), baseline to death (HR: 1.11, 95% CI: 1.06–1.16), and AF to complications (HR: 1.52, 95% CI: 1.37–1.68). In contrast, the Q4 of telomere length showed protective effects against transitions from baseline to AF (HR: 0.83, 95% CI: 0.80–0.86), baseline to death (HR: 0.91, 95% CI: 0.88–0.94), and AF to complication (HR: 0.78, 95% CI: 0.72–0.84).

**Table 4.** **Multistate model to assess associations of biological ageing with AF and its state transitions**.

Exposure	Model		HR (95% CI)				
			Baseline to AF	Baseline to Death	AF to Complication	AF to Death	Complication to Death
KDM-BA							
	Model 1						
		Per score	1.01 (1.01, 1.01) *	1.01 (1.01, 1.01) *	1.02 (1.02, 1.02) *	1.01 (1.01, 1.01) *	1.00 (0.99, 1.01)
		Q2	1.08 (1.04, 1.12) *	1.02 (0.97, 1.06)	1.41 (1.29, 1.53) *	1.00 (0.91, 1.1)	1.09 (0.82, 1.46)
		Q3	1.15 (1.10, 1.20) *	1.06 (1.01, 1.11) *	1.61 (1.46, 1.78) *	1.05 (0.94, 1.16)	1.08 (0.78, 1.50)
		Q4	1.27 (1.22, 1.33) *	1.12 (1.07, 1.18) *	2.21 (2.01, 2.44) *	1.02 (0.92, 1.14)	1.15 (0.83, 1.59)
	Model 2						
		Per score	1.01 (1.01, 1.01) *	1.01 (1.01, 1.01) *	1.02 (1.01, 1.02) *	1.01 (1.01, 1.01) *	1.00 (0.99, 1.01)
		Q2	1.01 (0.98, 1.05) *	1.01 (0.96, 1.05)	1.26 (1.16, 1.38) *	1.02 (0.93, 1.12)	1.09 (0.82, 1.46)
		Q3	1.04 (0.99, 1.08) *	1.04 (0.99, 1.10)	1.38 (1.25, 1.52) *	1.08 (0.97, 1.20)	1.06 (0.76, 1.48)
		Q4	1.09 (1.04, 1.14) *	1.10 (1.04, 1.16) *	1.75 (1.58, 1.94) *	1.08 (0.97, 1.21)	1.15 (0.82, 1.61)
PhenoAge							
	Model 1						
		Per score	1.03 (1.03, 1.03) *	1.01 (1.01, 1.02) *	1.04 (1.04, 1.05) *	0.99 (0.98, 1.00) *	1.00 (0.98, 1.02)
		Q2	1.12 (1.08, 1.16) *	1.02 (0.98, 1.06)	1.23 (1.11, 1.37) *	0.97 (0.88, 1.06)	0.97 (0.66, 1.41)
		Q3	1.19 (1.15, 1.24) *	1.03 (0.99, 1.08)	1.49 (1.35, 1.65) *	0.98 (0.89, 1.07)	0.97 (0.68, 1.39)
		Q4	1.43 (1.37, 1.48) *	1.12 (1.08, 1.17) *	1.86 (1.69, 2.05) *	0.93 (0.85, 1.02)	1.00 (0.71, 1.42)
	Model 2						
		Per score	1.02 (1.02, 1.03) *	1.01 (1.01, 1.02) *	1.03 (1.02, 1.04) *	0.99 (0.99, 1.00) *	1.00 (0.98, 1.02)
		Q2	1.08 (1.04, 1.12) *	1.02 (0.98, 1.06)	1.15 (1.04, 1.28) *	0.98 (0.90, 1.07)	0.95 (0.64, 1.39)
		Q3	1.12 (1.08, 1.16) *	1.03 (0.99, 1.07)	1.32 (1.19, 1.47) *	1.00 (0.91, 1.09)	0.94 (0.65, 1.36)
		Q4	1.30 (1.25, 1.35) *	1.11 (1.06, 1.16) *	1.52 (1.37, 1.68) *	0.96 (0.87, 1.05)	0.96 (0.67, 1.37)
Telomere							
	Model 1						
		Per score	0.93 (0.92, 0.94) *	0.96 (0.95, 0.97) *	0.89 (0.87, 0.91) *	1.02 (1.00, 1.05) *	0.99 (0.92, 1.06)
		Q2	0.92 (0.89, 0.94) *	0.92 (0.88, 0.95) *	0.89 (0.83, 0.95) *	1.00 (0.93, 1.07)	0.88 (0.72, 1.07)
		Q3	0.86 (0.83, 0.89) *	0.90 (0.87, 0.93) *	0.75 (0.70, 0.81) *	1.03 (0.96, 1.11)	0.87 (0.70, 1.08)
		Q4	0.79 (0.77, 0.82) *	0.88 (0.86, 0.92) *	0.72 (0.67, 0.78) *	1.06 (0.99, 1.14)	1.00 (0.80, 1.25)
	Model 2						
		Per score	0.94 (0.93, 0.95) *	0.97 (0.96, 0.98) *	0.91 (0.89, 0.94) *	1.02 (0.99, 1.04)	0.99 (0.92, 1.07)
		Q2	0.93 (0.90, 0.96) *	0.93 (0.90, 0.96) *	0.91 (0.85, 0.98) *	0.99 (0.93, 1.06)	0.89 (0.73, 1.08)
		Q3	0.89 (0.86, 0.92) *	0.91 (0.88, 0.94) *	0.80 (0.74, 0.86) *	1.02 (0.95, 1.09)	0.86 (0.69, 1.08)
		Q4	0.83 (0.80, 0.86) *	0.91 (0.88, 0.94) *	0.78 (0.72, 0.84) *	1.05 (0.97, 1.13)	1.02 (0.82, 1.28)

Model 1 was adjusted for sex and ethnicity; Model 2 was adjusted for sex, ethnicity, education, Townsend deprivation index, BMI, diet, smoking status, and alcohol intake. AF-related complications included heart failure, myocardial infarction, stroke, dementia and other arterial embolism diseases. *p < 0.05.

Further analysis of specific complications revealed distinct patterns of biological ageing markers in different transitions (Table 5, **Supplementary Table 5**). KDM-BA and PhenoAge showed significant associations with increased risks of transitions from baseline to AF (HR: 1.09, 95% CI: 1.04–1.14 for KDM-BA Q4; HR: 1.30, 95% CI: 1.25–1.35 for PhenoAge Q4), baseline to death (HR: 1.10, 95% CI: 1.04–1.16 for KDM-BA Q4; HR: 1.11, 95% CI: 1.06–1.16 for PhenoAge Q4), and AF to HF (HR: 1.89, 95% CI: 1.65–2.17 for KDM-BA Q4; HR: 1.60, 95% CI: 1.40–1.83 for PhenoAge Q4). Additionally, PhenoAge was associated with increased risks of transitions from AF to other arterial embolism diseases (HR: 1.49, 95% CI: 1.25–1.79 for Q4), and MI to death (HR: 2.53, 95% CI: 1.44–4.45 for Q4). In contrast, telomere length demonstrated protective effects against the progression from baseline to AF (HR: 0.83, 95% CI: 0.80–0.86 for Q4), baseline to death (HR: 0.91, 95% CI: 0.88–0.94 for Q4), and AF to specific complications, especially in reducing the risk of transitions to MI (HR: 0.57, 95% CI: 0.49–0.67), dementia (HR: 0.62, 95% CI: 0.47–0.81), and other arterial embolism diseases (HR: 0.84, 95% CI: 0.73–0.96).

**Table 5.** **Details of the multistate model assessment of the associations of biological ageing with AF and its state transitions in Model 2**.

Exposure	State transition	HR (95% CI)			
		Per score	Q2	Q3	Q4
KDM-BA					
	Health to AF	1.01 (1.01, 1.01) *	1.01 (0.98, 1.05)	1.04 (0.99, 1.08)	1.09 (1.04, 1.14) *
	Health to death	1.01 (1.01, 1.01) *	1.01 (0.96, 1.05)	1.04 (0.99, 1.10)	1.10 (1.04, 1.16) *
	AF to HF	1.02 (1.01, 1.02) *	1.25 (1.11, 1.41) *	1.49 (1.30, 1.70) *	1.89 (1.65, 2.17) *
	AF to MI	1.00 (0.99, 1.02)	1.13 (0.94, 1.36)	0.98 (0.80, 1.21)	1.12 (0.90, 1.41)
	AF to dementia	1.01 (1.00, 1.02) *	0.92 (0.66, 1.28)	1.18 (0.82, 1.70)	0.91 (0.39, 2.16)
	AF to other arterial embolism diseases	1.01 (1.01, 1.02) *	1.26 (1.07, 1.47) *	1.27 (1.06, 1.53) *	0.96 (0.58, 1.59)
	AF to death	1.01 (1.01, 1.01) *	1.02 (0.93, 1.12)	1.08 (0.97, 1.20)	1.08 (0.97, 1.21)
	HF to death	1.02 (0.99, 1.06)	0.91 (0.23, 3.59)	3.29 (0.97, 11.17)	2.74 (0.78, 9.65)
	MI to death	1.02 (1.01, 1.04) *	6.65 (0.00, 12.72)	7.21 (0.00, 19.72)	9.99 (0.00, 25.06)
	Dementia to death	1.01 (1.00, 1.02) *	1.35 (0.57, 3.15)	1.17 (0.80, 1.73)	1.13 (0.43, 2.95)
	Other arterial embolism diseases to death	1.00 (0.99, 1.01)	0.88 (0.58, 1.35)	0.91 (0.56, 1.49)	0.96 (0.58, 1.59)
PhenoAge					
	Health to AF	1.02 (1.02, 1.03) *	1.08 (1.04, 1.12) *	1.12 (1.08, 1.16) *	1.30 (1.25, 1.35) *
	Health to death	1.01 (1.01, 1.02) *	1.02 (0.98, 1.06)	1.03 (0.99, 1.07)	1.11 (1.06, 1.16) *
	AF to HF	1.04 (1.03, 1.04) *	1.14 (0.99, 1.32)	1.25 (1.09, 1.44) *	1.60 (1.40, 1.83) *
	AF to MI	0.99 (0.98, 1.01)	0.93 (0.78, 1.12)	0.93 (0.78, 1.12)	0.93 (0.77, 1.11)
	AF to dementia	1.01 (0.99, 1.03)	1.12 (0.80, 1.58)	1.03 (0.73, 1.47)	1.09 (0.77, 1.53)
	AF to other arterial embolism diseases	1.00 (0.99, 1.02)	1.05 (0.86, 1.27)	1.36 (1.13, 1.64) *	1.49 (1.25, 1.79) *
	AF to death	0.99 (0.99, 1.00)	0.98 (0.90, 1.07)	1.00 (0.91, 1.09)	0.96 (0.87, 1.05)
	HF to death	0.98 (0.92, 1.06)	0.57 (0.09, 3.69)	0.45 (0.09, 2.15)	0.46 (0.10, 2.13)
	MI to death	1.06 (1.02, 1.10) *	1.47 (0.87, 2.69)	0.98 (0.52, 1.85)	2.53 (1.44, 4.45) *
	Dementia to death	1.00 (0.94, 1.05)	1.38 (0.58, 3.30)	1.95 (0.80, 4.71)	1.03 (0.41, 2.55)
	Other arterial embolism diseases to death	1.04 (1.01, 1.08) *	0.77 (0.44, 1.35)	0.90 (0.54, 1.51)	0.85 (0.52, 1.40)
Telomere					
	Health to AF	0.94 (0.93, 0.95) *	0.93 (0.90, 0.96) *	0.89 (0.86, 0.92) *	0.83 (0.80, 0.86) *
	Health to death	0.97 (0.96, 0.98) *	0.93 (0.90, 0.96) *	0.91 (0.88, 0.94) *	0.91 (0.88, 0.94) *
	AF to HF	0.62 (0.46, 0.82) *	0.95 (0.87, 1.04)	0.85 (0.77, 0.94) *	1.42 (0.63, 3.19)
	AF to MI	0.18 (0.12, 0.28) *	0.83 (0.73, 0.94) *	0.69 (0.60, 0.80) *	0.57 (0.49, 0.67) *
	AF to dementia	0.16 (0.08, 0.34) *	0.92 (0.74, 1.15)	0.78 (0.61, 1.00) *	0.62 (0.47, 0.81) *
	AF to other arterial embolism diseases	0.45 (0.31, 0.66) *	0.90 (0.80, 1.02)	0.81 (0.71, 0.93) *	0.84 (0.73, 0.96) *
	AF to death	1.02 (0.99, 1.04)	1.02 (0.99, 1.04)	1.02 (0.99, 1.04)	1.02 (0.99, 1.04)
	HF to death	2.09 (0.17, 26.09)	0.56 (0.26, 1.21)	0.81 (0.73, 0.90) *	0.46 (0.10, 2.13)
	MI to death	0.77 (0.16, 3.75)	0.72 (0.45, 1.17)	0.74 (0.45, 1.23)	0.95 (0.55, 1.64)
	Dementia to death	1.33 (0.46, 3.86)	0.93 (0.66, 1.30)	1.09 (0.77, 1.56)	1.26 (0.87, 1.83)
	Other arterial embolism diseases to death	0.65 (0.25, 1.67)	0.95 (0.71, 1.26)	0.80 (0.58, 1.12)	0.79 (0.56, 1.10)

Model 2 was adjusted for sex, ethnicity, education, Townsend deprivation index, BMI, diet, smoking status, and alcohol intake. Complications included heart failure, myocardial infarction, stroke, dementia and other arterial embolism diseases. *p < 0.05.

### 3.4 Mediation Analyses

**Fig. 4.**
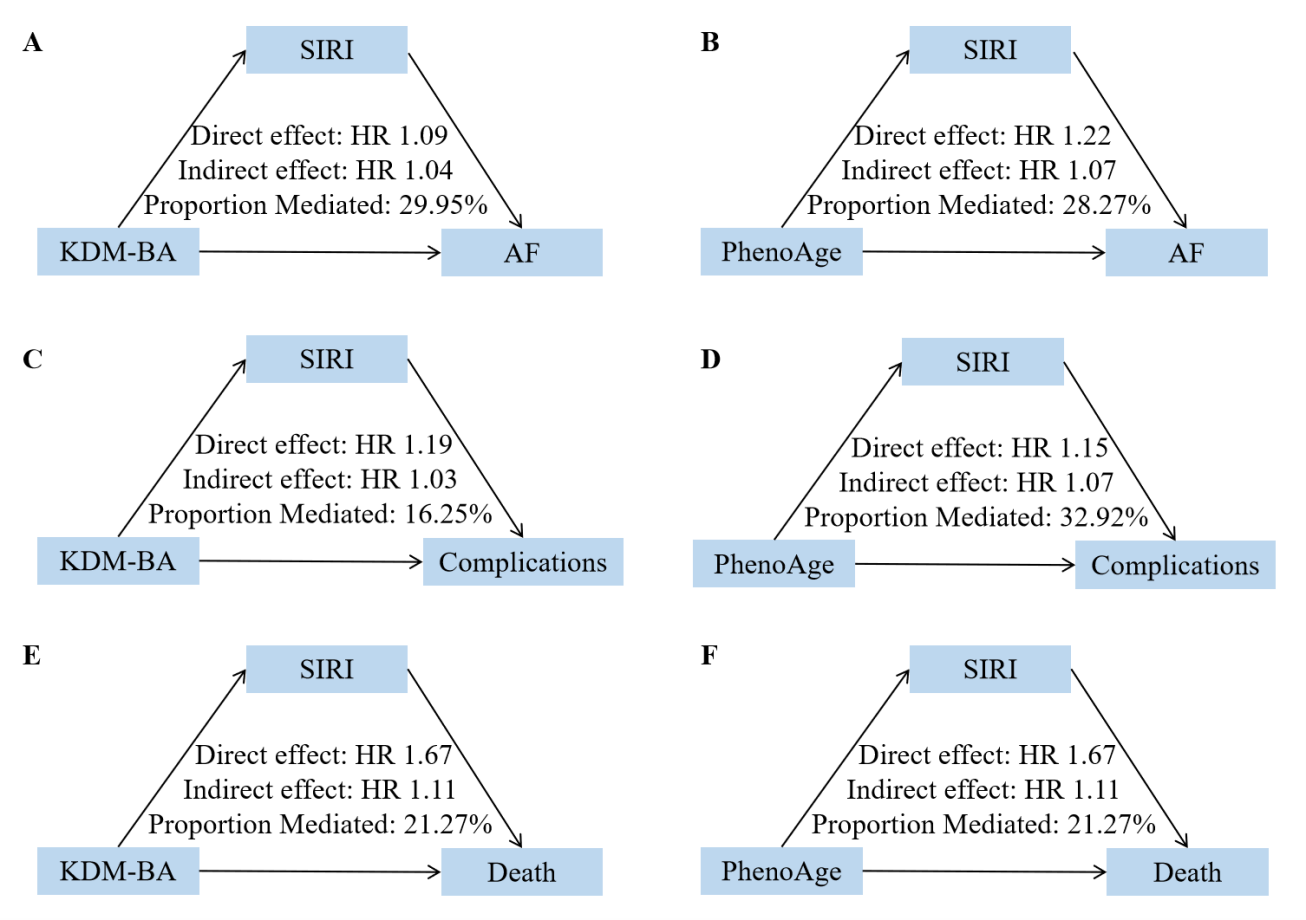
**Mediating role of SIRI in the associations of biological ageing with AF and its state transitions**. (A) Mediating role of SIRI in the associations of KDM-BA with AF. (B) Mediating role of SIRI in the associations of PhenoAge with AF. (C) Mediating role of SIRI in the associations of KDM-BA with complications. (D) Mediating role of SIRI in the associations of PhenoAge with complications. (E) Mediating role of SIRI in the associations of KDM-BA with death. (F) Mediating role of SIRI in the associations of PhenoAge with death. Complications included heart failure, myocardial infarction, stroke, dementia, and other arterial embolism diseases. SIRI, Systemic inflammatory response index.

Mediation analyses were performed to explore whether systemic inflammation mediated the associations between biological ageing markers and AF-related outcomes (Fig. 4, **Supplementary Table 6**). For both KDM-BA and PhenoAge, SIRI showed stronger mediating effects compared to NLR across all transitions. Specifically, SIRI mediated 29.95% and 28.27% of the total effects of KDM-BA and PhenoAge on AF incidence, respectively. For progression to complications, SIRI mediated 16.25% of the KDM-BA effect and 32.92% of the PhenoAge effect. The mediating role of SIRI was also evident in mortality transitions, accounting for 21.27% of the total effects for both KDM-BA and PhenoAge.

### 3.5 Sensitivity Analyses

Sensitivity analyses confirmed the robustness of the findings. Firstly, the analysis results after excluding the missing covariates were consistent (**Supplementary Table 7**), and the basic characteristics of the included and excluded populations seemed to have no difference (**Supplementary Tables 8,9**). Excluding events within the first two years of follow-up, participants with baseline cancer history, or employing alternative scales for biological ageing markers did not alter the primary results (**Supplementary Tables 10–12**). MSM results were re-estimated using a semi-Markov specification (time since entry into the current state as the time scale), yielding consistent estimates. Additionally, the analysis results were consistent using a semi-Markov specification with time-since-entry, and setting a prior state’s entry time to “±1–2 days” for same-day events (**Supplementary Tables 13–17**).

## 4. Discussion

Using a prospective cohort from the UK Biobank, this study is the first to comprehensively evaluate the associations between biological ageing markers and the dynamic progression of AF from a healthy state to AF onset, and subsequent AF-related complications. Our findings demonstrate that accelerated biological ageing, indicated by higher KDM-BA and PhenoAge, and shorter telomere length was significantly associated with increased risks across these transitions, particularly in increasing AF onset and progression to AF-related complications. Additionally, systemic inflammation, particularly SIRI, mediated these associations. These results underscore the importance of biological ageing as a key factor in the AF trajectory and highlight potential intervention targets to mitigate adverse outcomes.

Our findings are consistent with and build upon previous research into biological ageing and cardiovascular disease, particularly AF. Previous studies have consistently demonstrated that accelerated biological ageing, as measured by KDM-BA and PhenoAge, is a predictor of an increased risk of AF and related complications, supporting their utility as predictive markers in clinical practice [14, 23]. However, most previous studies have focused on the static associations between ageing markers and AF incidence. By incorporating MSMs, our study uniquely captures the dynamic progression of AF from a healthy state through to complications and ultimately mortality, thereby providing a more comprehensive understanding of disease trajectories. Our findings are consistent with those of He *et al*. [24], who also emphasized the role of biological ageing in cardiometabolic multimorbidity and mortality. Interestingly, our study reveals the distinct impact of KDM-BA, PhenoAge, and telomere length on various disease transitions. While Staerk *et al*. [25] and Siland *et al*. [26]. reported no clear association between telomere length and AF incidence, our results suggest that longer telomeres protect against progression to complications. These discrepancies may be due to differences in study design, population characteristics, or analytical methods, indicating a need for further research into telomere biology in AF pathogenesis. Overall, our findings corroborate the role of biological ageing in AF while offering novel insights into its dynamic effects across disease states.

Biological ageing is a key risk factor for AF, influencing its onset and progression through interconnected biological mechanisms. Structural and electrical remodeling of the atria, including age-related fibrosis and upregulation of matrix metalloproteinases, disrupts myocardial conduction and promotes arrhythmogenesis [27, 28]. Oxidative stress and mitochondrial dysfunction further exacerbate AF susceptibility by impairing mitochondrial function and calcium homeostasis, thereby contributing to atrial myopathy [29, 30]. Chronic low-grade inflammation also plays a central role by activating NF-κB signaling and inflammasome pathways, thereby driving fibrosis and the release of pro-inflammatory cytokines [31, 32]. Our mediation analysis underscores the role of systemic inflammation, specifically SIRI and the NLR, as intermediaries in linking biological ageing to AF onset and progression. Epigenetic modifications, including telomere shortening and DNA methylation, also contribute to AF by inducing cellular senescence, apoptosis, and fibrotic remodeling. Shortened telomeres, in particular, heighten susceptibility to AF through these pathways [33, 34]. DNA methylation, histone modifications, and non-coding RNA promote age-related atrial remodeling and arrhythmias by regulating gene expression and cell signaling pathways [35]. Biological ageing may increase the risk of AF through interrelated mechanisms involving atrial remodeling, mitochondrial dysfunction, chronic inflammation, and epigenetic alterations.

Biological ageing also accelerates the progression of AF-related complications and mortality by promoting systemic inflammation and mitochondrial dysfunction. Chronic inflammation, driven by elevated cytokines such as TNF-α and IL-6, promotes endothelial dysfunction, myocardial fibrosis, and vascular remodeling, thereby increasing the risk of heart failure, stroke, and dementia [36, 37]. Our mediation analysis reinforces the central role of systemic inflammatory markers such as SIRI and NLR in linking ageing to AF complications. Concurrently, mitochondrial dysfunction increases oxidative stress and impairs energy production, exacerbating cardiac and vascular damage, particularly in ischaemic conditions [38, 39]. Impaired mitophagy further amplifies these effects by failing to clear damaged mitochondria, and thus aggravating tissue injury. Epigenetic changes, including telomere shortening and DNA methylation, contribute to cellular senescence, neuroinflammation, and vascular pathology, thereby intensifying the risk of stroke and dementia [37, 40]. Biological ageing may drive AF complications and mortality through inflammation, mitochondrial dysfunction, and epigenetic alterations, providing potential therapeutic targets.

This study provides important clinical and public health insights by demonstrating the value of biological ageing markers in predicting the dynamic progression of AF. KDM-BA, PhenoAge, and telomere length effectively identify individuals at high risk for AF onset and progression to complications. Integrating these markers into clinical practice may be helpful for the early detection of AF progression. Public health efforts can benefit from understanding the relationship between ageing and AF state transitions, enabling early detection and monitoring of AF progression through ageing markers [41, 42]. Meanwhile, anti-ageing strategies may potentially reduce the incidence of early-onset AF and its complications [43]. These findings emphasize the need for ageing-focused strategies in personalized care and health policy to manage AF more effectively.

This study has several strengths. Firstly, by leveraging the large-scale UK Biobank cohort, which underwent comprehensive biological ageing assessments and long-term follow-up, we were able to systematically evaluate the role of ageing markers in AF progression using MSMs. This approach provided more accurate estimates than conventional methods. Secondly, we revealed the distinct impacts of KDM-BA, PhenoAge, and telomere length on AF progression, offering new insights into the differential roles of ageing markers. Thirdly, mediation analysis identified systemic inflammation as a key pathway linking biological ageing to AF outcomes, thereby reinforcing the mechanistic relevance of biological ageing.

### Limitations

However, several limitations should be acknowledged. First, as an observational study, causality cannot be established between biological ageing and AF progression. Second, baseline measurements of biological ageing markers and inflammatory indicators do not capture their dynamic changes over time. Third, although the proportion of systemic inflammatory mediators is as high as 30%, the interpretation of the results requires caution, and other mechanisms warrant further investigation. Fourth, the predominantly White and relatively healthier UK Biobank may lead to a bias in the health volunteers, thereby limiting the generalizability of the findings to other ethnic groups or less healthy populations. Fifth, we primarily identified new-onset AF and its complications through hospitalization and cause-of-death data. AF cases diagnosed in the community may have been missed. Such a delayed/latent AF diagnosis may introduce bias into state transitions and transition timing. Finally, despite comprehensive covariate adjustments, residual confounding remains plausible for unmeasured factors such as genetic predisposition, medication use (particularly anticoagulants, antihypertensives), and detailed cardiovascular risk factors. Future research should address these limitations by using repeated measurements, expanding to diverse and less healthy populations, and employing comprehensive data sources to capture all AF cases, thus enhancing robustness and generalizability.

## 5. Conclusions

In conclusion, this prospective study demonstrates that biological ageing markers are differentially associated with AF progression trajectories: accelerated ageing (indicated by elevated KDM-BA and PhenoAge, and shortened telomere length) primarily increases risks of AF onset and progression to complications, with systemic inflammation serving as a key mediator. These findings suggest that monitoring biological ageing markers and targeting anti-ageing-related pathways may be valuable strategies for preventing AF onset and its subsequent complications.

## Availability of Data and Materials

The data are available from the UK Biobank, but there are restrictions on their availability. Researchers who wish to access the UK Biobank database will need to apply for access through the following link: https://www.ukbiobank.ac.uk/enable-your-research/.

## Supplementary Material

Supplementary material associated with this article can be found, in the online version, at https://doi.org/10.31083/RCM47208.
