# Supplementary material for: Contributions of Biological Aging to Longitudinal Incidence and Dynamic Progression of Atrial Fibrillation: A Prospective Cohort Study
Source: Rev Cardiovasc Med. 2026 Mar 6;27(3):47208. doi: 10.31083/RCM47208 (PMC13036544; doi:10.31083/RCM47208)
Supplement: Supplementary file 1 [file 2153-8174-27-3-47208-s1.zip › Supplementary Material.doc]

Supplement

Supplementary Table 1 PH diagnostics for the associations of KDM-BA with AF and its state transition	2

Supplementary Table 2 PH diagnostics for the associations of PhenoAge with AF and its state transition	2

Supplementary Table 3 PH diagnostics for the associations of Telomere with AF and its state transition	3

Supplementary Table 4 Baseline characteristics of study participants by incident disease states in the data set of telomeres as exposure	4

Supplementary Table 5 Details of the multistate model assessment of the associations of biological ageing with AF and its state transitions in details in Model 1	5

Supplementary Table 6 Mediating role of inflammation in the associations of biological ageing with AF and its state transition	7

Supplementary Table 7 Sensitively analysis of multistate model to assess associations of biological ageing with AF and its state transition after excluding the population lacking covariates.	8

Supplementary Table 8 Baseline characteristics of study participants of exclude and include participants according to the missing covariates in the data set of KDM-BA and PhenoAge as exposure	9

Supplementary Table 9 Baseline characteristics of study participants of exclude and include participants according to the missing covariates in the data set of telomeres as exposure	9

Supplementary Table 10 Sensitively analysis of multistate model to assess associations of biological ageing with AF and its state transition after excluding the population occurred outcomes within two years	11

Supplementary Table 11 Sensitively analysis of multistate model to assess associations of biological ageing with AF and its state transition after excluding the population with history of cancer at baseline	12

Supplementary Table 12 Sensitively analysis of multistate model to assess associations of biological ageing with AF and its state transition using scale of age	13

Supplementary Table 13 Sensitively analysis of multistate model to assess associations of biological ageing with AF and its state transition using a semi-Markov specification (time since entry into the current state as the time scale).	14

Supplementary Table 14 Sensitively analysis of multistate model to assess associations of biological ageing with AF and its state transition setting a prior state’s entry time to ±1 days for same-day events	15

Supplementary Table 15 Sensitively analysis of multistate model to assess associations of biological ageing with AF and its state transition setting a prior state’s entry time to -1 days for same-day events	16

Supplementary Table 16 Sensitively analysis of multistate model to assess associations of biological ageing with AF and its state transition setting a prior state’s entry time to +2 days for same-day events	17

Supplementary Table 17 Sensitively analysis of multistate model to assess associations of biological ageing with AF and its state transition setting a prior state’s entry time to -2 days for same-day events.	18

**Supplementary Table 1 PH diagnostics for the associations of KDM-BA with AF and its state transition**

| **Outcome** | **Exposure** | **chisq** | **df** | ***P*** |
| --- | --- | --- | --- | --- |
| AF |  |  |  |  |
|  | KDM-BA | 2.372 | 3 | 0.499 |
|  | Sex | 8.701 | 1 | 0.003 |
|  | Townsend deprivation index | 6.97 | 1 | 0.008 |
|  | Ethnic | 4.283 | 1 | 0.039 |
|  | Education | 5.04 | 6 | 0.539 |
|  | BMI (kg/ m^2^) | 6.234 | 3 | 0.101 |
|  | Diet | 1.323 | 1 | 0.25 |
|  | Alcohol intake | 0.492 | 2 | 0.782 |
|  | Smoking status | 3.061 | 2 | 0.216 |
|  | GLOBAL | 39.785 | 20 | 0.005 |
| Complications |  |  |  |  |
|  | KDM-BA | 1.063 | 3 | 0.786 |
|  | Sex | 9.186 | 1 | 0.002 |
|  | Townsend deprivation index | 1.162 | 1 | 0.281 |
|  | Ethnic | 0.009 | 1 | 0.923 |
|  | Education | 9.322 | 6 | 0.156 |
|  | BMI (kg/ m^2^) | 1.675 | 3 | 0.642 |
|  | Diet | 0.025 | 1 | 0.874 |
|  | Alcohol intake | 2.096 | 2 | 0.351 |
|  | Smoking status | 8.682 | 2 | 0.013 |
|  | GLOBAL | 38.598 | 20 | 0.007 |
| Death |  |  |  |  |
|  | KDM-BA | 4.806 | 3 | 0.187 |
|  | Sex | 0.219 | 1 | 0.639 |
|  | Townsend deprivation index | 0.024 | 1 | 0.877 |
|  | Ethnic | 0.096 | 1 | 0.757 |
|  | Education | 2.119 | 6 | 0.908 |
|  | BMI (kg/ m^2^) | 0.342 | 3 | 0.952 |
|  | Diet | 0.316 | 1 | 0.574 |
|  | Alcohol intake | 0.221 | 2 | 0.895 |
|  | Smoking status | 1.712 | 2 | 0.425 |
|  | GLOBAL | 11.556 | 20 | 0.931 |

AF: Atrial fibrillation; BMI: Body mass index; KDM-BA: Klemera–Doubal method biological age.

**Supplementary Table 2 PH diagnostics for the associations of PhenoAge** with AF and its state transition

| **Outcome** | **Exposure** | **chisq** | **df** | ***P*** |
| --- | --- | --- | --- | --- |
| AF |  |  |  |  |
|  | PhenoAge | 14.286 | 3 | 0.003 |
|  | Sex | 8.558 | 1 | 0.003 |
|  | Townsend deprivation index | 6.99 | 1 | 0.008 |
|  | Ethnic | 4.282 | 1 | 0.039 |
|  | Education | 5.077 | 6 | 0.534 |
|  | BMI (kg/ m^2^) | 6.219 | 3 | 0.101 |
|  | Diet | 1.342 | 1 | 0.247 |
|  | Alcohol intake | 0.479 | 2 | 0.787 |
|  | Smoking status | 3.061 | 2 | 0.216 |
|  | GLOBAL | 50.034 | 20 | 0 |
| Complications |  |  |  |  |
|  | PhenoAge | 9.552 | 3 | 0.023 |
|  | Sex | 8.935 | 1 | 0.003 |
|  | Townsend deprivation index | 1.258 | 1 | 0.262 |
|  | Ethnic | 0.001 | 1 | 0.971 |
|  | Education | 9.537 | 6 | 0.146 |
|  | BMI (kg/ m^2^) | 1.694 | 3 | 0.638 |
|  | Diet | 0.016 | 1 | 0.899 |
|  | Alcohol intake | 2.002 | 2 | 0.367 |
|  | Smoking status | 8.758 | 2 | 0.013 |
|  | GLOBAL | 43.195 | 20 | 0.002 |
| Death |  |  |  |  |
|  | PhenoAge | 3.259 | 3 | 0.353 |
|  | Sex | 0.254 | 1 | 0.614 |
|  | Townsend deprivation index | 0.022 | 1 | 0.881 |
|  | Ethnic | 0.094 | 1 | 0.759 |
|  | Education | 2.004 | 6 | 0.919 |
|  | BMI (kg/ m^2^) | 0.297 | 3 | 0.961 |
|  | Diet | 0.373 | 1 | 0.541 |
|  | Alcohol intake | 0.217 | 2 | 0.897 |
|  | Smoking status | 1.672 | 2 | 0.433 |
|  | GLOBAL | 10.168 | 20 | 0.965 |

AF: Atrial fibrillation; BMI: Body mass index.

**Supplementary Table 3 PH diagnostics for the associations of Telomere** with AF and its state transition

| **Outcome** | **Exposure** | **chisq** | **df** | ***P*** |
| --- | --- | --- | --- | --- |
| AF |  |  |  |  |
|  | Telomere | 2.578 | 3 | 0.461 |
|  | Sex | 16.071 | 1 | 0 |
|  | Townsend deprivation index | 2.924 | 1 | 0.087 |
|  | Ethnic | 0.033 | 1 | 0.855 |
|  | Education | 1.658 | 6 | 0.948 |
|  | BMI (kg/ m^2^) | 9.468 | 3 | 0.024 |
|  | Diet | 0.968 | 1 | 0.325 |
|  | Alcohol intake | 1.407 | 2 | 0.495 |
|  | Smoking status | 5.867 | 2 | 0.053 |
|  | GLOBAL | 42.031 | 20 | 0.003 |
| Complications |  |  |  |  |
|  | Telomere | 1.904 | 3 | 0.593 |
|  | Sex | 11.658 | 1 | 0.001 |
|  | Townsend deprivation index | 0.158 | 1 | 0.691 |
|  | Ethnic | 1.763 | 1 | 0.184 |
|  | Education | 10.175 | 6 | 0.117 |
|  | BMI (kg/ m^2^) | 1.796 | 3 | 0.616 |
|  | Diet | 0.1 | 1 | 0.752 |
|  | Alcohol intake | 8.252 | 2 | 0.016 |
|  | Smoking status | 8.077 | 2 | 0.018 |
|  | GLOBAL | 44.927 | 20 | 0.001 |
| Death |  |  |  |  |
|  | Telomere | 5.214 | 3 | 0.157 |
|  | Sex | 0.073 | 1 | 0.787 |
|  | Townsend deprivation index | 0.305 | 1 | 0.581 |
|  | Ethnic | 1.531 | 1 | 0.216 |
|  | Education | 1.068 | 6 | 0.983 |
|  | BMI (kg/ m^2^) | 0.466 | 3 | 0.926 |
|  | Diet | 0.673 | 1 | 0.412 |
|  | Alcohol intake | 4.051 | 2 | 0.132 |
|  | Smoking status | 2.215 | 2 | 0.33 |
|  | GLOBAL | 17.855 | 20 | 0.597 |

AF: Atrial fibrillation; BMI: Body mass index.

**Supplementary Table 4** Baseline characteristics of study participants by incident disease states in the data set of telomeres as exposure

| **Characteristics** | **Overall**  **(n=** **339603)** | **AF**  **(n=** **32853)** | **AF-related complications**  **(n=** **5864)** |
| --- | --- | --- | --- |
| Age (years) | 55.58±8.06 | 56.85±8.05 | 58.19±7.99 |
| Sex |  |  |  |
| Female | 194446(57.26) | 15681(47.73) | 2228(37.99) |
| Male | 145157(42.74) | 17172(52.27) | 3636(62.01) |
| Ethnic |  |  |  |
| White | 320577(94.40) | 31482(95.83) | 5662(96.56) |
| Other | 19026(5.60) | 1371(4.17) | 202(3.44) |
| Education |  |  |  |
| College or university degree | 119632(35.23) | 10620(32.33) | 1523(25.97) |
| A/AS level or equivalent | 40044(11.79) | 3559(10.83) | 515(8.78) |
| O/GCSEs level or equivalent | 73590(21.67) | 6788(20.66) | 1196(20.40) |
| CSEs or equivalent | 18924(5.57) | 1487(4.53) | 204(3.48) |
| NVQ/HND/HNC or equivalent | 21052(6.20) | 2316(7.05) | 506(8.63) |
| Other professional qualifications | 17101(5.04) | 1786(5.44) | 338(5.76) |
| None of the above | 49260(14.51) | 6297(19.17) | 1582(26.98) |
| Townsend deprivation index | -1.45±3.01 | -1.48±3.01 | -1.31±3.09 |
| BMI (kg/ m^2^) | 26.91±4.43 | 27.70±4.78 | 28.90±5.19 |
| <18.5 | 2904(0.86) | 271(0.82) | 55(0.94) |
| 18.5-24.9 | 121053(35.65) | 9732(29.62) | 1250(21.32) |
| 25-29.9 | 145106(42.73) | 14234(43.33) | 2554(43.55) |
| ≥ 30 | 70540(20.77) | 8616(26.23) | 2005(34.19) |
| Diet |  |  |  |
| Healthy | 176262(51.90) | 17254(52.52) | 3053(52.06) |
| Unhealthy | 163341(48.10) | 15599(47.48) | 2811(47.94) |
| Smoking status |  |  |  |
| Never | 194602(57.30) | 17222(52.42) | 2669(45.52) |
| Previous | 112105(33.01) | 12324(37.51) | 2500(42.63) |
| Current | 32896(9.69) | 3307(10.07) | 695(11.85) |
| Alcohol intake |  |  |  |
| Never | 13754(4.05) | 1290(3.93) | 243(4.14) |
| Previous | 10332(3.04) | 1147(3.49) | 234(3.99) |
| Current | 315517(92.91) | 30416(92.58) | 5387(91.87) |
| Telomere | 0.84±0.13 | 0.82±0.13 | 0.81±0.13 |
| Q1 | 84900(25.00) | 9360(28.49) | 1957(33.37) |
| Q2 | 84900(25.00) | 8482(25.82) | 1613(27.51) |
| Q3 | 84899(25.00) | 7875(23.97) | 1235(21.06) |
| Q4 | 84904(25.00) | 7136(21.72) | 1059(18.06) |

AF-related complications included heart failure, myocardial infarction, stroke, dementia and other arterial embolism diseases. AF: Atrial fibrillation; BMI: Body mass index; KDM-BA: Klemera–Doubal method biological age.

**Supplementary Table 5** Details of the multistate model assessment of the associations of biological ageing with AF and its state transitions in details in Model 1

| **Exposure** | **State transition** | **HR(95%CI)** | | | |
| --- | --- | --- | --- | --- | --- |
|  |  | **Perscore** | **Q2** | **Q3** | **Q4** |
| **KDM-BA** | |  |  |  |  |
|  | Health to AF | 1.01(1.01,1.01)* | 1.08(1.04,1.12)* | 1.15(1.1,1.2)* | 1.27(1.22,1.33)* |
|  | Health to death | 1.01(1.01,1.01)* | 1.02(0.97,1.06) | 1.06(1.01,1.11)* | 1.12(1.07,1.18)* |
|  | AF to HF | 1.03(1.02,1.03)* | 1.45(1.29,1.63)* | 1.83(1.6,2.08)* | 2.56(2.25,2.91)* |
|  | AF to MI | 1(0.99,1.01) | 0.97(0.81,1.16) | 1.03(0.85,1.26) | 1.00(0.81,1.23) |
|  | AF to dementia | 1.01(1,1.02)* | 0.88(0.64,1.2) | 1.21(0.87,1.68) | 1.31(0.93,1.84) |
|  | AF to other arterial embolism diseases | 1.02(1.01,1.02)* | 1.37(1.17,1.6)* | 1.43(1.2,1.71)* | 1.99(1.67,2.38)* |
|  | AF to death | 1.01(1.01,1.01)* | 1(0.91,1.1) | 1.05(0.94,1.16) | 1.02(0.92,1.14) |
|  | HF to death | 1.01(0.99,1.04) | 1(0.29,3.49) | 2.37(0.78,7.24) | 2.01(0.72,5.65) |
|  | MI to death | 1.02(1.01,1.03)* | 1.42(0.89,2.27) | 1.30(0.75,2.26) | 1.85(1.04,3.28)* |
|  | Dementia to death | 0.99(0.97,1.01) | 0.92(0.44,1.92) | 0.61(0.28,1.32) | 0.89(0.39,2.02) |
|  | Other arterial embolism diseases to death | 1(0.99,1.01) | 0.87(0.57,1.32) | 0.91(0.56,1.47) | 0.91(0.56,1.47) |
| **PhenoAge** | |  |  |  |  |
|  | Health to AF | 1.03(1.03,1.03)* | 1.12(1.08,1.16)* | 1.19(1.15,1.24)* | 1.43(1.37,1.48)* |
|  | Health to death | 1.01(1.01,1.02)* | 1.02(0.98,1.06) | 1.03(0.99,1.08) | 1.12(1.08,1.17)* |
|  | AF to HF | 1.05(1.04,1.06)* | 1.24(1.07,1.44)* | 1.45(1.26,1.67)* | 2.08(1.83,2.37)* |
|  | AF to MI | 0.99(0.98,1.01) | 0.93(0.77,1.11) | 0.92(0.77,1.11) | 0.93(0.78,1.11) |
|  | AF to dementia | 1.02(1,1.04)* | 1.25(0.9,1.73) | 1.21(0.88,1.68) | 1.3(0.95,1.78) |
|  | AF to other arterial embolism diseases | 1.03(1.02,1.04)* | 1.12(0.92,1.36) | 1.51(1.26,1.82)* | 1.76(1.48,2.09)* |
|  | AF to death | 0.99(0.98,1) | 0.97(0.88,1.06) | 0.98(0.89,1.07) | 0.93(0.85,1.02) |
|  | HF to death | 0.99(0.93,1.05) | 0.41(0.08,2.06) | 0.48(0.12,1.88) | 0.48(0.14,1.62) |
|  | MI to death | 1.05(1.02,1.09)* | 1.44(0.80,2.60) | 1.01(0.55,1.87) | 2.22(1.30,3.79)* |
|  | Dementia to death | 0.99(0.94,1.04) | 1.45(0.64,3.28) | 1.73(0.78,3.86) | 1.11(0.5,2.49) |
|  | Other arterial embolism diseases to death | 1(0.97,1.03) | 0.78(0.45,1.35) | 0.9(0.55,1.49) | 0.86(0.53,1.39) |
| **Telomere** | |  |  |  |  |
|  | Health to AF | 0.93(0.92,0.94)* | 0.92(0.89,0.94)* | 0.86(0.83,0.89)* | 0.79(0.77,0.82)* |
|  | Health to death | 0.96(0.95,0.97)* | 0.92(0.88,0.95)* | 0.9(0.87,0.93)* | 0.88(0.86,0.92)* |
|  | AF to HF | 0.49(0.37,0.65)* | 0.93(0.85,1.02) | 0.8(0.72,0.88)* | 0.75(0.68,0.83)* |
|  | AF to MI | 0.18(0.12,0.28)* | 0.83(0.73,0.94)* | 0.69(0.60,0.79) * | 0.57(0.49,0.67)* |
|  | AF to dementia | 0.13(0.06,0.27)* | 0.9(0.72,1.12) | 0.74(0.58,0.94)* | 0.57(0.43,0.74)* |
|  | AF to other arterial embolism diseases | 0.45(0.31,0.67)* | 0.88(0.78,1) | 0.77(0.68,0.88)* | 0.78(0.68,0.89)* |
|  | AF to death | 1.02(1,1.05)* | 1(0.93,1.07) | 1.03(0.96,1.11) | 1.06(0.99,1.14) |
|  | HF to death | 0.58(0.05,6.94)* | 0.53(0.26,1.07) | 0.49(0.23,1.05) | 0.99(0.46,2.11) |
|  | MI to death | 1.32(0.45,3.84) | 0.95(0.68,1.33) | 1.11(0.78,1.57) | 1.23(0.85,1.78) |
|  | Dementia to death | 0.86(0.19,3.92) | 0.74(0.47,1.18) | 0.8(0.49,1.3) | 0.96(0.57,1.62) |
|  | Other arterial embolism diseases to death | 0.5(0.2,1.22) | 1(0.76,1.33) | 0.83(0.6,1.15) | 0.83(0.59,1.15) |

Model 1 was adjusted for sex and ethnic. Complications included heart failure, myocardial infarction, stroke, dementia and other arterial embolism diseases. AF: Atrial fibrillation; BMI: Body mass index; HF: Heart failure; MI: Myocardial infarction; KDM-BA: Klemera–Doubal method biological age. **P*<0.05.

**Supplementary Table 6 Mediating role of inflammation in the associations of biological ageing with AF and its state transition**

| **Outcome** | **Effect** | **KDM-BA** | | **PhenoAge** | | **Telomere** | |
| --- | --- | --- | --- | --- | --- | --- | --- |
|  |  | **NLR** | **SIRI** | **NLR** | **SIRI** | **NLR** | **SIRI** |
| **AF** |  |  |  |  |  |  |  |
|  | **Direct effect** | 1.13(1.10,1.16)* | 1.09(1.06,1.14)* | 1.27(1.12,1.43)* | 1.22(1.19,1.25)* | 0.76(0.75,0.79)* | 0.76(0.74,0.77)* |
|  | **Indirect effect** | 1.01(1.01,1.01)* | 1.04(1.03,1.04)* | 1.03(1.02,1.04)* | 1.07(1.06,1.08)* | 1.00(1.00,1.00) | 1.00(1.00,1.00) |
|  | **Total effect** | 1.14(1.09,1.24)* | 1.14(1.10,1.18)* | 1.31(1.26,1.37)* | 1.31(1.26,1.33)* | 0.76(0.74,0.79)* | 0.76(0.74,0.77)* |
|  | **Proportion Mediated (%)** | 9.29(6.48,13.27)* | 29.95(23.30,40.27)* | 12.94(8.55,15.74)* | 28.27(23.97,32.11)* | -0.40(-0.66,-0.15) | 0.12(-0.00,0.24) |
| **Complications** |  |  |  |  |  |  |  |
|  | **Direct effect** | 1.23(1.07,1.30)* | 1.19(1.08,1.20)* | 1.21(1.05,1.24)* | 1.15(1.11,1.23)* | 0.55(0.52,0.61)* | 0.55(0.51,0.58)* |
|  | **Indirect effect** | 1.01(1.00,1.01)* | 1.03(1.02,1.04)* | 1.02(0.99,1.04) | 1.07(1.05,1.10)* | 1.00(1.00,1.00) | 1.00(1.00,1.00) |
|  | **Total effect** | 1.23(1.10,1.35)* | 1.23(1.12,1.22)* | 1.23(1.10,1.32)* | 1.23(1.17,1.37)* | 0.55(0.50,0.61)* | 0.55(0.51,0.58)* |
|  | **Proportion Mediated (%)** | 3.59(0.70,7.21) | 16.25(9.09,31.51)* | 8.09(-2.88,26.50) | 32.92(24.75,45.07)* | -0.11(-0.21,-0.05) | 0.04(-0.05,0.13) |
| **Death** |  |  |  |  |  |  |  |
|  | **Direct effect** | 1.74(1.55,1.89)* | 1.67(1.62,1.74)* | 1.74(1.55,1.86)* | 1.67(1.58,1.73)* | 0.92(0.90,0.93)* | 0.92(0.88,0.93)* |
|  | **Indirect effect** | 1.07(1.06,1.08)* | 1.11(1.09,1.13)* | 1.07(1.06,1.09)* | 1.11(1.10,1.13)* | 1.00(1.00,1.00) | 1.00(1.00,1.00) |
|  | **Total effect** | 1.86(1.80,1.974)* | 1.85(1.78,1.92)* | 1.86(1.67,1.98)* | 1.85(1.77,1.90)* | 0.92(0.91,0.95)* | 0.92(0.88,0.93)* |
|  | **Proportion Mediated (%)** | 14.27(11.65,16.80)* | 21.27(19.19,24.23)* | 14.27(11.55,17.62)* | 21.27(19.60,25.25)* | -3.18(-5.97,-2.11) | 0.74(-0.18,1.02) |

Models were adjusted for sex, ethnic, education, Townsend deprivation index, BMI, diet, smoking status, and alcohol intake. The strata variable was not included in the model when stratifying by itself. Complications included heart failure, myocardial infarction, stroke, dementia and other arterial embolism diseases. AF: Atrial fibrillation; BMI: Body mass index; KDM-BA: Klemera–Doubal method biological age. **P*<0.05.

**Supplementary Table 7** Sensitively analysis of multistate model to assess associations of biological ageing with AF and its state transition after excluding the population lacking covariates.

| **Exposure** | **Model** | | **HR (95% CI)** | | | | |
| --- | --- | --- | --- | --- | --- | --- | --- |
|  |  |  | **Baseline to AF** | **Baseline to Death** | **AF to Complication** | **AF to Death** | **Complication to Death** |
| **KDM-BA** |  |  |  |  |  |  |  |
|  |  | Per score | 1.00(1.00,1.01)* | 1.02(1.01,1.03)* | 1.00(1.00,1.01)* | 1.01(1.01,1.02)* | 1.02(1.01,1.02)* |
|  |  | Q2 | 1.03(0.99,1.08) | 1.23(1.16,1.30)* | 1.11(1.02,1.22)* | 1.19(1.05,1.34)* | 1.52(1.12,2.08)* |
|  |  | Q3 | 1.07(1.03,1.13)* | 1.34(1.26,1.43)* | 1.09(0.98,1.21) | 1.31(1.15,1.50)* | 1.06(0.72,1.56) |
|  |  | Q4 | 1.16(1.10,1.21)* | 1.70(1.59,1.81)* | 1.12(1.00,1.25)* | 1.58(1.37,1.82)* | 1.64(1.12,2.41)* |
| **PhenoAge** |  |  |  |  |  |  |  |
|  |  | Per score | 1.03(1.02,1.03)* | 1.06(1.05,1.06)* | 1.00(0.99,1.00) | 1.05(1.05,1.06)* | 1.04(1.01,1.06)* |
|  |  | Q2 | 1.07(1.03,1.12)* | 1.12(1.05,1.19)* | 0.95(0.86,1.04) | 1.24(1.07,1.45)* | 1.25(0.85,1.83) |
|  |  | Q3 | 1.16(1.12,1.21)* | 1.30(1.22,1.38)* | 0.97(0.89,1.07) | 1.42(1.23,1.65)* | 1.27(0.87,1.86) |
|  |  | Q4 | 1.34(1.28,1.39)* | 1.79(1.70,1.90)* | 0.95(0.87,1.05) | 1.89(1.64,2.18)* | 1.67(1.16,2.40)* |
| **Telomere** |  |  |  |  |  |  |  |
|  |  | Per score | 0.44(0.40,0.48)* | 0.72(0.66,0.80)* | 0.34(0.28,0.42)* | 1.14(0.93,1.40) | 0.82(0.43,1.56) |
|  |  | Q2 | 0.91(0.88,0.94)* | 0.92(0.89,0.96)* | 0.87(0.82,0.94)* | 0.99(0.92,1.07) | 0.78(0.64,0.96)* |
|  |  | Q3 | 0.84(0.82,0.87)* | 0.91(0.88,0.94)* | 0.73(0.68,0.79)* | 1.03(0.95,1.11) | 0.82(0.66,1.02) |
|  |  | Q4 | 0.76(0.74,0.79)* | 0.90(0.87,0.94)* | 0.70(0.65,0.75)* | 1.05(0.97,1.13) | 1.01(0.81,1.25) |

Models were adjusted for sex, ethnic, education, Townsend deprivation index, BMI, diet, smoking status, and alcohol intake. The strata variable was not included in the model when stratifying by itself. Complications included heart failure, myocardial infarction, stroke, dementia and other arterial embolism diseases. AF: Atrial fibrillation; BMI: Body mass index; KDM-BA: Klemera–Doubal method biological age. **P*<0.05**.**

**Supplementary Table 8 Baseline characteristics of study participants of exclude and include participants according to the missing covariates in the data set of KDM-BA and PhenoAge as exposure**

| Characteristics | Exclude  (n= 20312) | Include  (n= 239886) |
| --- | --- | --- |
| Age (years) | 56.61±8.08 | 56.52±8.10 |
| Sex |  |  |
| Female | 11395(56.10) | 137137(57.17) |
| Male | 8917(43.90) | 102749(42.83) |
| Ethnic |  |  |
| White | 16913(84.20) | 228782(95.37) |
| Other | 3173(15.80) | 11104(4.63) |
| Education |  |  |
| College or university degree | 4109(23.23) | 86490(36.05) |
| A/AS level or equivalent | 1422(8.04) | 29224(12.18) |
| O/GCSEs level or equivalent | 3461(19.56) | 53330(22.23) |
| CSEs or equivalent | 1282(7.25) | 13524(5.64) |
| NVQ/HND/HNC or equivalent | 1387(7.84) | 14464(6.03) |
| Other professional qualifications | 909(5.14) | 11861(4.94) |
| None of the above | 5121(28.95) | 30993(12.92) |
| Townsend deprivation index | -0.35±3.45 | -1.59±2.91 |
| BMI (kg/ m^2^) | 27.32±4.64 | 26.83±4.35 |
| Thin(<18.5) | 532(2.62) | 1262(0.53) |
| Normal(18.5-24.9) | 6402(31.52) | 87387(36.43) |
| Overweight(25-29.9) | 8586(42.27) | 102984(42.93) |
| Obesity(≥ 30) | 4792(23.59) | 48253(20.11) |
| Diet |  |  |
| Healthy | 1640(54.70) | 124513(51.91) |
| Unhealthy | 1358(45.30) | 115373(48.09) |
| Smoking status |  |  |
| Never | 10997(57.32) | 138458(57.72) |
| Previous | 5768(30.07) | 79643(33.20) |
| Current | 2419(12.61) | 21785(9.08) |
| Alcohol intake |  |  |
| Never | 1862(9.42) | 8439(3.52) |
| Previous | 1035(5.23) | 6533(2.72) |
| Current | 16878(85.35) | 224914(93.76) |

AF: Atrial fibrillation; BMI: Body mass index; KDM-BA: Klemera–Doubal method biological age.

**Supplementary Table 9 Baseline characteristics of study participants of exclude and include participants according to the missing covariates in the data set of telomeres as exposure**

| Characteristics | Exclude  (n= 27848) | Include  (n= 311755) |
| --- | --- | --- |
| Age (years) | 56.51±8.11 | 56.53±8.09 |
| Sex |  |  |
| Female | 15528(55.76) | 178918(57.39) |
| Male | 12320(44.24) | 132837(42.61) |
| Ethnic |  |  |
| White | 23034(83.72) | 297223(95.34) |
| Other | 4479(16.28) | 14532(4.66) |
| Education |  |  |
| College or university degree | 5654(23.30) | 112737(36.16) |
| A/AS level or equivalent | 1951(8.04) | 37688(12.09) |
| O/GCSEs level or equivalent | 4648(19.15) | 68170(21.87) |
| CSEs or equivalent | 1658(6.83) | 17046(5.47) |
| NVQ/HND/HNC or equivalent | 1842(7.59) | 18995(6.09) |
| Other professional qualifications | 1339(5.52) | 15563(4.99) |
| None of the above | 7177(29.57) | 41556(13.33) |
| Townsend deprivation index | -0.26±3.50 | -1.55±2.94 |
| BMI (kg/ m^2^) | 27.44±4.74 | 26.88±4.40 |
| <18.5 | 1262(4.53) | 1642(0.53) |
| 18.5-24.9 | 8476(30.44) | 112577(36.11) |
| 25-29.9 | 11431(41.05) | 133675(42.88) |
| ≥ 30 | 6679(23.98) | 63861(20.48) |
| Diet |  |  |
| Healthy | 2389(54.20) | 161972(51.95) |
| Unhealthy | 2019(45.80) | 149783(48.05) |
| Smoking status |  |  |
| Never | 14867(56.53) | 178841(57.37) |
| Previous | 7976(30.33) | 103632(33.24) |
| Current | 3456(13.14) | 29282(9.39) |
| Alcohol intake |  |  |
| Never | 2618(9.68) | 11043(3.54) |
| Previous | 1476(5.46) | 8831(2.83) |
| Current | 22963(84.87) | 291881(93.63) |

AF: Atrial fibrillation; BMI: Body mass index.

**Supplementary Table 10** Sensitively analysis of multistate model to assess associations of biological ageing with AF and its state transition after excluding the population occurred outcomes within two years

| **Exposure** | **Model** | | **HR (95% CI)** | | | | |
| --- | --- | --- | --- | --- | --- | --- | --- |
|  |  |  | **Baseline to AF** | **Baseline to Death** | **AF to Complication** | **AF to Death** | **Complication to Death** |
| **KDM-BA** |  |  |  |  |  |  |  |
|  |  | Per score | 1.01(1.01,1.01)* | 1.01(1.01,1.01)* | 1.02(1.01,1.02)* | 1.01(1.01,1.01)* | 1(0.99,1.01) |
|  |  | Q2 | 1.01(0.98,1.05) | 1.02(0.97,1.06) | 1.27(1.16,1.38)* | 1.02(0.92,1.12) | 1.06(0.79,1.42) |
|  |  | Q3 | 1.03(0.99,1.08) | 1.04(0.99,1.1) | 1.38(1.25,1.53)* | 1.07(0.96,1.18) | 1.05(0.75,1.47) |
|  |  | Q4 | 1.08(1.03,1.13)* | 1.12(1.06,1.18)* | 1.75(1.58,1.94)* | 1.07(0.96,1.2) | 1.14(0.81,1.61) |
| **PhenoAge** |  |  |  |  |  |  |  |
|  |  | Per score | 1.02(1.02,1.03)* | 1.01(1.01,1.01)* | 1.03(1.02,1.04)* | 0.99(0.99,1) | 1(0.98,1.02) |
|  |  | Q2 | 1.08(1.04,1.12)* | 1.02(0.98,1.07) | 1.15(1.04,1.28)* | 0.99(0.9,1.08) | 0.94(0.64,1.39) |
|  |  | Q3 | 1.12(1.08,1.16)* | 1.02(0.98,1.07) | 1.32(1.19,1.47)* | 1(0.91,1.09) | 0.9(0.62,1.31) |
|  |  | Q4 | 1.3(1.25,1.35)* | 1.1(1.05,1.15)* | 1.52(1.38,1.68)* | 0.96(0.87,1.05) | 0.95(0.67,1.36) |
| **Telomere** |  |  |  |  |  |  |  |
|  |  | Per score | 0.56(0.51,0.61)* | 0.74(0.67,0.82)* | 0.49(0.4,0.61)* | 1.1(0.91,1.34) | 0.86(0.45,1.65) |
|  |  | Q2 | 0.93(0.9,0.96)* | 0.93(0.9,0.96)* | 0.92(0.86,0.98)* | 1(0.93,1.07) | 0.9(0.74,1.11) |
|  |  | Q3 | 0.89(0.86,0.91)* | 0.92(0.89,0.95)* | 0.8(0.75,0.86)* | 1.02(0.95,1.1) | 0.86(0.69,1.07) |
|  |  | Q4 | 0.83(0.8,0.85)* | 0.91(0.88,0.94)* | 0.78(0.72,0.84)* | 1.05(0.97,1.13) | 1.04(0.83,1.3) |

Models were adjusted for sex, ethnic, education, Townsend deprivation index, BMI, diet, smoking status, and alcohol intake. The strata variable was not included in the model when stratifying by itself. Complications included heart failure, myocardial infarction, stroke, dementia and other arterial embolism diseases. AF: Atrial fibrillation; BMI: Body mass index; KDM-BA: Klemera–Doubal method biological age. **P*<0.05.

**Supplementary Table 11** Sensitively analysis of multistate model to assess associations of biological ageing with AF and its state transition after excluding the population with history of cancer at baseline

| **Exposure** | **Model** | | **HR (95% CI)** | | | | |
| --- | --- | --- | --- | --- | --- | --- | --- |
|  |  |  | **Baseline to AF** | **Baseline to Death** | **AF to Complication** | **AF to Death** | **Complication to Death** |
| **KDM-BA** |  |  |  |  |  |  |  |
|  |  | Per score | 1.01(1.01,1.01)* | 1.01(1.01,1.01)* | 1.02(1.01,1.02)* | 1.01(1.01,1.01)* | 1(0.99,1.01) |
|  |  | Q2 | 1.02(0.98,1.06) | 1(0.95,1.05) | 1.26(1.15,1.38)* | 0.99(0.9,1.09) | 1.01(0.75,1.37) |
|  |  | Q3 | 1.04(1,1.09)* | 1.03(0.98,1.09) | 1.38(1.24,1.53)* | 1.07(0.96,1.19) | 1.06(0.75,1.5) |
|  |  | Q4 | 1.09(1.04,1.15)* | 1.08(1.03,1.15)* | 1.75(1.57,1.95)* | 1.06(0.95,1.2) | 0.97(0.68,1.39) |
| **PhenoAge** |  |  |  |  |  |  |  |
|  |  | Per score | 1.02(1.02,1.03)* | 1.01(1.01,1.01)* | 1.03(1.02,1.04)* | 0.99(0.99,1) | 1(0.98,1.02) |
|  |  | Q2 | 1.09(1.04,1.13)* | 1(0.96,1.05) | 1.18(1.06,1.32)* | 0.98(0.9,1.08) | 1(0.67,1.5) |
|  |  | Q3 | 1.13(1.08,1.17)* | 1.02(0.98,1.07) | 1.35(1.21,1.5)* | 1.01(0.92,1.11) | 1(0.68,1.47) |
|  |  | Q4 | 1.3(1.25,1.35)* | 1.08(1.04,1.13)* | 1.54(1.38,1.71)* | 0.96(0.87,1.06) | 0.97(0.66,1.41) |
| **Telomere** |  |  |  |  |  |  |  |
|  |  | Per score | 0.43(0.39,0.47)* | 0.71(0.64,0.79)* | 0.35(0.28,0.43)* | 1.17(0.94,1.47) | 0.74(0.37,1.46) |
|  |  | Q2 | 0.93(0.9,0.96)* | 0.93(0.9,0.96)* | 0.9(0.84,0.96)* | 1.01(0.94,1.09) | 0.88(0.71,1.09) |
|  |  | Q3 | 0.9(0.87,0.92)* | 0.92(0.88,0.95)* | 0.79(0.73,0.85)* | 1.04(0.96,1.12) | 0.82(0.65,1.04) |
|  |  | Q4 | 0.83(0.8,0.86)* | 0.91(0.88,0.95)* | 0.76(0.7,0.83)* | 1.07(0.99,1.16) | 1.01(0.8,1.28) |

Models were adjusted for sex, ethnic, education, Townsend deprivation index, BMI, diet, smoking status, and alcohol intake. The strata variable was not included in the model when stratifying by itself. Complications included heart failure, myocardial infarction, stroke, dementia and other arterial embolism diseases. AF: Atrial fibrillation; BMI: Body mass index; KDM-BA: Klemera–Doubal method biological age. **P*<0.05.

**Supplementary Table 12** Sensitively analysis of multistate model to assess associations of biological ageing with AF and its state transition using scale of age

| **Exposure** | **Model** | | **HR (95% CI)** | | | | |
| --- | --- | --- | --- | --- | --- | --- | --- |
|  |  |  | **Baseline to AF** | **Baseline to Death** | **AF to Complication** | **AF to Death** | **Complication to Death** |
| **KDM-BA** |  |  |  |  |  |  |  |
|  |  | Per score | 1.01(1.01,1.01)* | 1.01(1.01,1.01)* | 1.02(1.01,1.02)* | 1.01(1.01,1.01)* | 1(0.99,1.01) |
|  |  | Q2 | 1.01(0.97,1.05) | 1.02(0.97,1.07) | 1.27(1.16,1.38)* | 1.01(0.92,1.11) | 1.05(0.78,1.41) |
|  |  | Q3 | 1.03(0.99,1.08) | 1.04(0.98,1.09) | 1.38(1.25,1.53)* | 1.05(0.95,1.17) | 1.04(0.74,1.45) |
|  |  | Q4 | 1.08(1.03,1.13)* | 1.1(1.04,1.17)* | 1.76(1.58,1.95)* | 1.07(0.95,1.2) | 1.13(0.8,1.59) |
| **PhenoAge** |  |  |  |  |  |  |  |
|  |  | Per score | 1.02(1.02,1.03)* | 1.01(1.01,1.01)* | 1.03(1.02,1.04)* | 0.99(0.99,1) | 1(0.98,1.02) |
|  |  | Q2 | 1.08(1.04,1.12)* | 1.02(0.98,1.07) | 1.16(1.04,1.29)* | 0.99(0.91,1.09) | 0.97(0.65,1.43) |
|  |  | Q3 | 1.12(1.08,1.16)* | 1.02(0.97,1.06) | 1.33(1.2,1.47)* | 1.01(0.92,1.11) | 0.94(0.64,1.37) |
|  |  | Q4 | 1.3(1.25,1.35)* | 1.1(1.05,1.15)* | 1.52(1.38,1.68)* | 0.96(0.87,1.05) | 0.98(0.68,1.41) |
| **Telomere** |  |  |  |  |  |  |  |
|  |  | Per score | 0.56(0.51,0.61)* | 0.75(0.68,0.83)* | 0.5(0.4,0.62)* | 1.11(0.91,1.35) | 0.92(0.48,1.75) |
|  |  | Q2 | 0.93(0.9,0.96)* | 0.94(0.9,0.97)* | 0.92(0.86,0.98)* | 1(0.93,1.07) | 0.91(0.75,1.12) |
|  |  | Q3 | 0.89(0.86,0.91)* | 0.92(0.89,0.95)* | 0.8(0.75,0.87)* | 1.02(0.95,1.1) | 0.87(0.7,1.09) |
|  |  | Q4 | 0.83(0.8,0.85)* | 0.91(0.88,0.94)* | 0.78(0.73,0.85)* | 1.05(0.97,1.13) | 1.06(0.84,1.33) |

Models were adjusted for sex, ethnic, education, Townsend deprivation index, BMI, diet, smoking status, and alcohol intake. The strata variable was not included in the model when stratifying by itself. Complications included heart failure, myocardial infarction, stroke, dementia and other arterial embolism diseases. AF: Atrial fibrillation; BMI: Body mass index; KDM-BA: Klemera–Doubal method biological age. **P*<0.05.

**Supplementary Table 13 Sensitively analysis of multistate model to assess associations of biological ageing with AF and its state transition using a semi-Markov specification (time since entry into the current state as the time scale)**.

| **Exposure** | **Model** | | **HR (95% CI)** | | | | |
| --- | --- | --- | --- | --- | --- | --- | --- |
|  |  |  | **Baseline to AF** | **Baseline to Death** | **AF to Complication** | **AF to Death** | **Complication to Death** |
| **KDM-BA** |  |  |  |  |  |  |  |
|  |  | Per score | 1.01(1.01,1.01)* | 1.01(1.01,1.01)* | 1.01(1.01,1.02)* | 1(0.99,1) | 1(0.99,1.01) |
|  |  | Q2 | 1(0.96,1.03) | 0.99(0.95,1.04) | 1.27(1.17,1.39)* | 0.98(0.89,1.08) | 1.3(0.95,1.78) |
|  |  | Q3 | 0.99(0.95,1.04) | 1(0.95,1.06) | 1.26(1.14,1.39)* | 0.95(0.86,1.06) | 1.56(1.07,2.27)* |
|  |  | Q4 | 1(0.96,1.05) | 1.03(0.97,1.08) | 1.63(1.47,1.81)* | 0.96(0.86,1.07) | 1.3(0.87,1.93) |
| **PhenoAge** |  |  |  |  |  |  |  |
|  |  | Per score | 1.01(1,1.01)* | 1(0.99,1) | 1.03(1.02,1.03)* | 0.99(0.98,1) | 1.01(0.99,1.04) |
|  |  | Q2 | 1.03(0.99,1.07) | 0.97(0.93,1.01) | 1.17(1.05,1.3)* | 0.98(0.89,1.07) | 1(0.65,1.52) |
|  |  | Q3 | 1.05(1.01,1.09)* | 0.97(0.93,1.01) | 1.25(1.13,1.39)* | 0.91(0.83,1) | 1.13(0.75,1.69) |
|  |  | Q4 | 1.09(1.05,1.13)* | 0.94(0.91,0.99) | 1.45(1.31,1.61)* | 0.91(0.83,1) | 1.03(0.7,1.53) |
| **Telomere** |  |  |  |  |  |  |  |
|  |  | Per score | 0.98(0.97,0.99)* | 1(0.99,1.01) | 0.91(0.88,0.93)* | 1.01(0.98,1.03) | 1.07(0.98,1.16) |
|  |  | Q2 | 0.99(0.96,1.02) | 0.98(0.94,1.01) | 0.96(0.9,1.03) | 1.06(0.99,1.14) | 1.1(0.89,1.37) |
|  |  | Q3 | 0.97(0.94,1) | 0.99(0.95,1.02) | 0.79(0.73,0.85)* | 1.01(0.94,1.09) | 1.13(0.89,1.43) |
|  |  | Q4 | 0.93(0.9,0.96)* | 1.01(0.97,1.04) | 0.77(0.71,0.83)* | 1.04(0.97,1.12) | 1.26(0.99,1.6) |

Models were adjusted for sex, ethnic, education, Townsend deprivation index, BMI, diet, smoking status, and alcohol intake. The strata variable was not included in the model when stratifying by itself. Complications included heart failure, myocardial infarction, stroke, dementia and other arterial embolism diseases. AF: Atrial fibrillation; BMI: Body mass index; KDM-BA: Klemera–Doubal method biological age. **P*<0.05.

**Supplementary Table 14** Sensitively analysis of multistate model to assess associations of biological ageing with AF and its state transition setting a prior state’s entry time to ±1 days for same-day events

| **Exposure** | **Model** | | **HR (95% CI)** | | | | |
| --- | --- | --- | --- | --- | --- | --- | --- |
|  |  |  | **Baseline to AF** | **Baseline to Death** | **AF to Complication** | **AF to Death** | **Complication to Death** |
| **KDM-BA** |  |  |  |  |  |  |  |
|  |  | Per score | 1.01(1.01,1.01)* | 1.02(1.01,1.02)* | 1.01(1.01,1.02)* | 1.01(1.01,1.02)* | 1(1,1.01)* |
|  |  | Q2 | 1.02(0.97,1.07) | 1.25(1.18,1.32)* | 1.32(1.2,1.45)* | 1.15(1,1.34)* | 0.93(0.75,1.15) |
|  |  | Q3 | 1.06(1,1.12)* | 1.34(1.25,1.43)* | 1.37(1.22,1.53)* | 1.25(1.05,1.48)* | 1.05(0.82,1.34) |
|  |  | Q4 | 1.16(1.09,1.23)* | 1.71(1.59,1.83)* | 1.71(1.52,1.93)* | 1.49(1.25,1.78)* | 1.08(0.85,1.38) |
| **PhenoAge** |  |  |  |  |  |  |  |
|  |  | Per score | 1.04(1.04,1.04)* | 1.06(1.05,1.06)* | 1.02(1.01,1.03)* | 1.05(1.04,1.05)* | 1.01(1,1.03)* |
|  |  | Q2 | 1.16(1.09,1.22)* | 1.12(1.05,1.19)* | 1.1(0.98,1.23) | 1.36(1.11,1.65)* | 1.02(0.77,1.35) |
|  |  | Q3 | 1.28(1.21,1.35)* | 1.32(1.24,1.41)* | 1.17(1.05,1.31)* | 1.38(1.14,1.68)* | 0.89(0.68,1.16) |
|  |  | Q4 | 1.57(1.49,1.66)* | 1.83(1.72,1.94)* | 1.29(1.15,1.44)* | 1.89(1.57,2.27)* | 1.09(0.84,1.4) |
| **Telomere** |  |  |  |  |  |  |  |
|  |  | Per score | 0.36(0.32,0.4)* | 0.3(0.26,0.33)* | 0.69(0.55,0.87)* | 0.4(0.29,0.55)* | 0.66(0.41,1.05) |
|  |  | Q2 | 0.86(0.82,0.89)* | 0.83(0.8,0.87)* | 0.97(0.9,1.04)* | 0.85(0.76,0.95)* | 0.86(0.74,1.01) |
|  |  | Q3 | 0.79(0.76,0.83)* | 0.77(0.74,0.81)* | 0.88(0.81,0.95)* | 0.78(0.69,0.87)* | 0.85(0.72,1.01) |
|  |  | Q4 | 0.71(0.68,0.74)* | 0.69(0.66,0.73)* | 0.92(0.85,1)* | 0.81(0.72,0.92)* | 0.93(0.78,1.11) |

Models were adjusted for sex, ethnic, education, Townsend deprivation index, BMI, diet, smoking status, and alcohol intake. The strata variable was not included in the model when stratifying by itself. Complications included heart failure, myocardial infarction, stroke, dementia and other arterial embolism diseases. AF: Atrial fibrillation; BMI: Body mass index; KDM-BA: Klemera–Doubal method biological age. **P*<0.05.

**Supplementary Table 15** Sensitively analysis of multistate model to assess associations of biological ageing with AF and its state transition setting a prior state’s entry time to -1 days for same-day events

| **Exposure** | **Model** | | **HR (95% CI)** | | | | |
| --- | --- | --- | --- | --- | --- | --- | --- |
|  |  |  | **Baseline to AF** | **Baseline to Death** | **AF to Complication** | **AF to Death** | **Complication to Death** |
| **KDM-BA** |  |  |  |  |  |  |  |
|  |  | Per score | 1.01(1.01,1.01)* | 1.02(1.01,1.02)* | 1.01(1.01,1.02)* | 1.01(1.01,1.02)* | 1(1,1.01)* |
|  |  | Q2 | 1.02(0.97,1.07) | 1.25(1.18,1.32)* | 1.32(1.2,1.45)* | 1.15(1,1.34)* | 0.93(0.75,1.15) |
|  |  | Q3 | 1.06(1,1.12)* | 1.34(1.25,1.43)* | 1.37(1.22,1.53)* | 1.25(1.05,1.48)* | 1.05(0.82,1.34) |
|  |  | Q4 | 1.16(1.09,1.23)* | 1.71(1.59,1.83)* | 1.71(1.52,1.93)* | 1.49(1.25,1.78)* | 1.08(0.85,1.38) |
| **PhenoAge** |  |  |  |  |  |  |  |
|  |  | Per score | 1.04(1.04,1.04)* | 1.06(1.05,1.06)* | 1.02(1.01,1.03)* | 1.05(1.04,1.05)* | 1.01(1,1.03)* |
|  |  | Q2 | 1.16(1.09,1.22)* | 1.12(1.05,1.19)* | 1.1(0.98,1.23) | 1.36(1.11,1.65)* | 1.02(0.77,1.35) |
|  |  | Q3 | 1.28(1.21,1.35)* | 1.32(1.24,1.41)* | 1.17(1.05,1.31)* | 1.38(1.14,1.68)* | 0.89(0.68,1.16) |
|  |  | Q4 | 1.57(1.49,1.66)* | 1.83(1.72,1.94)* | 1.29(1.15,1.44)* | 1.89(1.57,2.27)* | 1.09(0.84,1.4) |
| **Telomere** |  |  |  |  |  |  |  |
|  |  | Per score | 0.36(0.32,0.4)* | 0.3(0.26,0.33)* | 0.69(0.55,0.87)* | 0.4(0.29,0.55)* | 0.66(0.41,1.05) |
|  |  | Q2 | 0.86(0.82,0.89)* | 0.83(0.8,0.87)* | 0.97(0.9,1.04) | 0.85(0.76,0.95)* | 0.86(0.74,1.01) |
|  |  | Q3 | 0.79(0.76,0.83)* | 0.77(0.74,0.81)* | 0.88(0.81,0.95)* | 0.78(0.69,0.87)* | 0.85(0.72,1.01) |
|  |  | Q4 | 0.71(0.68,0.74)* | 0.69(0.66,0.73)* | 0.92(0.85,1)* | 0.81(0.72,0.92)* | 0.93(0.78,1.11) |

Models were adjusted for sex, ethnic, education, Townsend deprivation index, BMI, diet, smoking status, and alcohol intake. The strata variable was not included in the model when stratifying by itself. Complications included heart failure, myocardial infarction, stroke, dementia and other arterial embolism diseases. AF: Atrial fibrillation; BMI: Body mass index; KDM-BA: Klemera–Doubal method biological age. **P*<0.05.

**Supplementary Table 16** Sensitively analysis of multistate model to assess associations of biological ageing with AF and its state transition setting a prior state’s entry time to +2 days for same-day events

| **Exposure** | **Model** | | **HR (95% CI)** | | | | |
| --- | --- | --- | --- | --- | --- | --- | --- |
|  |  |  | **Baseline to AF** | **Baseline to Death** | **AF to Complication** | **AF to Death** | **Complication to Death** |
| **KDM-BA** |  |  |  |  |  |  |  |
|  |  | Per score | 1.01(1.01,1.01)* | 1.02(1.01,1.02)* | 1.01(1.01,1.02)* | 1.01(1.01,1.02)* | 1(1,1.01)* |
|  |  | Q2 | 1.02(0.97,1.07) | 1.25(1.18,1.32)* | 1.32(1.2,1.45)* | 1.15(1,1.34)* | 0.93(0.75,1.15) |
|  |  | Q3 | 1.06(1,1.12)* | 1.34(1.25,1.43)* | 1.37(1.22,1.53)* | 1.25(1.05,1.48)* | 1.05(0.82,1.34) |
|  |  | Q4 | 1.16(1.09,1.23)* | 1.71(1.59,1.83)* | 1.71(1.52,1.93)* | 1.49(1.25,1.78)* | 1.08(0.85,1.38) |
| **PhenoAge** |  |  |  |  |  |  |  |
|  |  | Per score | 1.04(1.04,1.04)* | 1.06(1.05,1.06)* | 1.02(1.01,1.03)* | 1.05(1.04,1.05)* | 1.01(1,1.03)* |
|  |  | Q2 | 1.16(1.09,1.22)* | 1.12(1.05,1.19)* | 1.1(0.98,1.23) | 1.36(1.11,1.65)* | 1.02(0.77,1.35) |
|  |  | Q3 | 1.28(1.21,1.35)* | 1.32(1.24,1.41)* | 1.17(1.05,1.31)* | 1.38(1.14,1.68)* | 0.89(0.68,1.16) |
|  |  | Q4 | 1.57(1.49,1.66)* | 1.83(1.72,1.94)* | 1.29(1.15,1.44)* | 1.89(1.57,2.27)* | 1.09(0.84,1.4) |
| **Telomere** |  |  |  |  |  |  |  |
|  |  | Per score | 0.36(0.32,0.4)* | 0.3(0.26,0.33)* | 0.69(0.55,0.87)* | 0.4(0.29,0.55)* | 0.66(0.41,1.05) |
|  |  | Q2 | 0.86(0.83,0.9)* | 0.82(0.79,0.85)* | 0.95(0.88,1.02) | 0.87(0.79,0.96)* | 0.88(0.76,1.02) |
|  |  | Q3 | 0.8(0.77,0.83)* | 0.76(0.73,0.79)* | 0.86(0.8,0.93)* | 0.77(0.69,0.85)* | 0.84(0.71,0.98)* |
|  |  | Q4 | 0.71(0.68,0.74)* | 0.68(0.65,0.71)* | 0.89(0.82,0.97)* | 0.8(0.72,0.9)* | 0.9(0.76,1.07) |

Models were adjusted for sex, ethnic, education, Townsend deprivation index, BMI, diet, smoking status, and alcohol intake. The strata variable was not included in the model when stratifying by itself. Complications included heart failure, myocardial infarction, stroke, dementia and other arterial embolism diseases. AF: Atrial fibrillation; BMI: Body mass index; KDM-BA: Klemera–Doubal method biological age. **P*<0.05.

**Supplementary Table 17** Sensitively analysis of multistate model to assess associations of biological ageing with AF and its state transition setting a prior state’s entry time to -2 days for same-day events.

| **Exposure** | **Model** | | **HR (95% CI)** | | | | |
| --- | --- | --- | --- | --- | --- | --- | --- |
|  |  |  | **Baseline to AF** | **Baseline to Death** | **AF to Complication** | **AF to Death** | **Complication to Death** |
| **KDM-BA** |  |  |  |  |  |  |  |
|  |  | Per score | 1.01(1.01,1.01)* | 1.02(1.01,1.02)* | 1.01(1.01,1.02)* | 1.01(1.01,1.02)* | 1(1,1.01) |
|  |  | Q2 | 1.02(0.97,1.07) | 1.25(1.18,1.32)* | 1.32(1.2,1.45)* | 1.15(1,1.34)* | 0.93(0.75,1.15) |
|  |  | Q3 | 1.06(1,1.12)* | 1.34(1.25,1.43)* | 1.37(1.22,1.53)* | 1.25(1.05,1.48)* | 1.05(0.82,1.34) |
|  |  | Q4 | 1.16(1.09,1.23)* | 1.71(1.59,1.83)* | 1.71(1.52,1.93)* | 1.49(1.25,1.78)* | 1.08(0.85,1.38) |
| **PhenoAge** |  |  |  |  |  |  |  |
|  |  | Per score | 1.04(1.04,1.04)* | 1.06(1.05,1.06)* | 1.02(1.01,1.03)* | 1.05(1.04,1.05)* | 1.01(1,1.03)* |
|  |  | Q2 | 1.16(1.09,1.22)* | 1.12(1.05,1.19)* | 1.1(0.98,1.23) | 1.36(1.11,1.65)* | 1.02(0.77,1.35) |
|  |  | Q3 | 1.28(1.21,1.35)* | 1.32(1.24,1.41)* | 1.17(1.05,1.31)* | 1.38(1.14,1.68)* | 0.89(0.68,1.16) |
|  |  | Q4 | 1.57(1.49,1.66)* | 1.83(1.72,1.94)* | 1.29(1.15,1.44)* | 1.89(1.57,2.27)* | 1.09(0.84,1.4) |
| **Telomere** |  |  |  |  |  |  |  |
|  |  | Per score | 0.36(0.32,0.4)* | 0.3(0.26,0.33)* | 0.69(0.55,0.87)* | 0.4(0.29,0.55)* | 0.66(0.41,1.05) |
|  |  | Q2 | 0.86(0.83,0.9)* | 0.82(0.79,0.85)* | 0.95(0.88,1.02) | 0.87(0.79,0.96)* | 0.88(0.76,1.02) |
|  |  | Q3 | 0.8(0.77,0.83)* | 0.76(0.73,0.79)* | 0.86(0.8,0.93)* | 0.77(0.69,0.85)* | 0.84(0.71,0.98)* |
|  |  | Q4 | 0.71(0.68,0.74)* | 0.68(0.65,0.71)* | 0.89(0.82,0.97)* | 0.8(0.72,0.9)* | 0.9(0.76,1.07) |

Models were adjusted for sex, ethnic, education, Townsend deprivation index, BMI, diet, smoking status, and alcohol intake. The strata variable was not included in the model when stratifying by itself. Complications included heart failure, myocardial infarction, stroke, dementia and other arterial embolism diseases. AF: Atrial fibrillation; BMI: Body mass index; KDM-BA: Klemera–Doubal method biological age. **P*<0.05.
